# Demonstrating and disrupting well-learned habits

**DOI:** 10.1371/journal.pone.0234424

**Published:** 2020-06-12

**Authors:** Ahmet O. Ceceli, Catherine E. Myers, Elizabeth Tricomi

**Affiliations:** 1 Department of Psychology, Rutgers University-Newark, Newark, NJ, United States of America; 2 Neurobehavioral Research Laboratory, VA New Jersey Health Care System, East Orange, NJ, United States of America; 3 Department of Pharmacology, Physiology & Neuroscience, New Jersey Medical School, Rutgers University, Newark, NJ, United States of America; Universiteit Gent, BELGIUM

## Abstract

Researchers have exerted tremendous efforts to empirically study how habits form and dominate at the expense of deliberation, yet we know very little about breaking these rigid habits to restore goal-directed control. In a three-experiment study, we first illustrate a novel approach of studying well-learned habits, in order to effectively demonstrate habit disruption. In Experiment 1, we use a Go/NoGo task with familiar color–response associations to demonstrate outcome-insensitivity when compared to novel, more flexible associations. Specifically, subjects perform more accurately when the required mapping is the familiar association of green–Go/red–NoGo than when it is red–Go/green–NoGo, confirming outcome-insensitive, habitual control. As a control condition, subjects show equivalent performance with unfamiliar color–response mappings (using the colors blue and purple mapped to Go and NoGo responses). Next, in Experiments 2 and 3, we test a motivation-based feedback manipulation in varying magnitudes (i.e., performance feedback with and without monetary incentives) to break the well-established habits elicited by our familiar stimuli. We find that although performance feedback prior to the contingency reversal test is insufficient to disrupt outcome-insensitivity in Experiment 2, a combination of performance feedback and monetary incentive is able to restore goal-directed control in Experiment 3, effectively breaking the habits. As the first successful demonstration of well-learned habit disruption in the laboratory, these findings provide new insights into how we execute and modify habits, while fostering new and translational research avenues that may be applicable to treating habit-based pathologies.

## Introduction

When categorizing motivated behaviors, habits are distinguished from goal-directed actions in that they are performed reflexively in response to a triggering cue (i.e., governed by a stimulus), without consideration of the consequences [1]. These habitual behaviors are less cognitively taxing than their goal-directed counterparts, allowing for their utilization in instances where the resource-consuming reflection of potential outcomes may not be ideal [2–4]. For example, looking both ways before crossing a street is an action best elicited habitually, and ideally should persist despite the absence of oncoming traffic. In contrast, the optimal motivational control system for commuting to a new destination would be the outcome-reliant and resource-consuming goal-directed performance.

For decades, the motivational bases of behavioral control (i.e., goal-directed and habitual actions) have been investigated in rodent models. In a typical study examining habitual control, a neutral stimulus (e.g., a visual cue, or the context of the chamber) signals hungry rats to press a lever in pursuit of a food outcome. This behavioral training period is often followed by a devaluation procedure—the rat is allowed free-access to the food, promoting satiation and diminishing the food’s value (hence the term *devaluation*). In a subsequent, unrewarded, extinction phase, the experimenter can then assess whether the trained lever-press action is flexible and goal-directed (i.e., strong responses when animal is hungry but diminished responses when satiated), or rigid and habitual (i.e., persistent responses regardless of satiation) [5]. Generally, over-training of the stimulus—response—outcome association tends to render actions habitual [6]. Thus, an over-trained rat persists in pressing the lever despite a diminished value in outcome, suggesting that the actions are driven by the preceding cue or the chamber context. In contrast, value-driven goal-directed control survives following moderate experience with the stimulus–response–outcome contingencies [6]. Habit testing in humans has followed suit with similar operant conditioning paradigms, in which a primary or a secondary reward is devalued to determine whether actions are cue or value driven [7–12]. Another widely-used example is the sequential decision task, in which subjects respond to probabilistic multi-step associative sequences and recruit model-based (i.e., goal-directed; taking into account the cognitive model of the task environment) or model-free (i.e., similar to habits; actions based solely on history of reward receipt) strategies to maximize gain and minimize loss [13].

These methods have undoubtedly contributed a great deal to our understanding of habits; however, such paradigms are limited in critical aspects. Indeed, the habit experience has been a difficult construct to effectively capture via behavioral paradigms in humans [14–16]. First, in contemporary paradigms, including those based on outcome-devaluation and sequential decision-making, the agent must develop a newly formed habit. Accordingly, the tools at our disposal facilitate the study of novel, lab-developed habits, while leaving incomplete our understanding of well-learned habits that are more representative of daily experiences. For example, especially in outcome-devaluation tasks involving valued and devalued food rewards, testing whether a behavior is habitual relies on several critical factors. The demonstration of a habit may depend on successful over-training of a new stimulus–response–outcome association that develops a strong enough link between the stimulus and the response to guide behavior [11]. Furthermore, the effectiveness of the devaluation procedure where a food outcome is selectively fed to diminish its value may become problematic in humans for reasons not encountered in rats, such as demand characteristics and hesitation to eat copious amounts of junk food in a potentially socially intimidating lab setting. Lastly, the experimenter makes assumptions of comparable food palatability, in that the agent must value the food options similarly prior to selective devaluation for any value-based manipulation to be effective [11]. These lab-generated habits are also arduous to develop via over-training, especially in expensive neuroimaging contexts. More importantly, the strength of the trained habit would be insufficient for a meaningful investigation of the habit-breaking process, in that even multi-day training is often measured in minutes to hours [11,17]. Thus, the current tools provide a costly platform that only captures the unidirectional shift from goal-directed to habitual control [18]. In other words, although these novel, lab-created associations permit the study of habit formation and execution, we are limited in our tools to investigate habit disruption with similar efficacy.

Accessing the shift from habitual to goal-directed control may ultimately facilitate interventions that remediate rigid and maladaptive behaviors, yet we are not currently methodologically equipped to tackle this translational research avenue with a rich toolkit. Accordingly, we propose that developing a novel habit from an action–outcome contingency is not a pre-requisite for studying the motivational basis for habits, but that an existing, more robust habit could be examined in the lab with less effort. An effective approach may involve using salient cues that elicit well-established, habit-like behaviors that are impervious to their consequences. For instance, the colors red and green have highly specific “stop” and “go” associations, possibly strengthened in a variety of contexts including traffic lights, visual signals of danger and safety, and childhood games, songs, and stories [19]. The familiar red–stop and green–go contingencies have previously been transformed into Go/NoGo and stop-signal tasks to assess response inhibition via perseverative errors (i.e., NoGo accuracy) [19–22]. Similarly, we can test for behavioral rigidity by assessing performance when these contingencies are congruent with daily experiences versus when adjusted to reflect outcomes incongruent with most real-world scenarios. Thus, instead of devaluing the palatability of a primary reward, we render a well-learned association inappropriate for optimal task performance. The agent must override a prepotent red stimulus–stop response with an incongruent green stimulus–stop response to achieve the intended, correct outcome. A more pronounced accuracy change when managing incongruencies within this well-learned color–response mapping, compared to changes in a newly-acquired mapping, would permit us to conclude that these familiar stimuli evoke outcome-insensitive actions, the hallmark of habitual behavior. Upon establishing that these familiar stimuli elicit habitual control, we can then provide the platform to study habit disruption by testing manipulations that protect against mapping-related performance changes–essentially overriding the habitual response by engaging cognitive control processes. The motivational control framework identifies habits as cue-dependent, and goal-directed behaviors as those contingent on the outcome (please refer to [1,18,23] for a more thorough description of motivation as it relates to habits and goal-directed behaviors). Accordingly, a previously goal-directed behavior is rendered habitual when the associative strength of the stimulus–response component governs actions, rendering the outcome inessential for action execution. A promising strategy for restoring goal-directed control may be via boosting the salience of the outcome—for instance, by enhancing the link between the response and outcome.

Providing opportunities for performance tracking and administering other forms of performance-based feedback (e.g., primary and secondary rewards) have been used extensively in enhancing behavioral output [24,25]. For instance, the delivery of performance tracking information combined with a monetary reward successfully improved performance on a visual task [25]. A combination of primary and secondary rewards (e.g., juice and monetary incentives) has also been documented to improve goal-directed performance on a cued task-switching paradigm via motivational enhancement [26]. The promise of a future reward contingent on performance has sufficed in improving performance during task-switching, and accelerating responses during a reaction time task with congruent and incongruent stimuli [27,28]. Furthermore, trial-by-trial, transient monetary incentives (i.e., increasing reward magnitudes from low to high across trials) have served as salient performance boosters in tasks that taxed executive control, as well as visual perception [29]. Taken together with the finding that performance-contingent monetary rewards engage top-down control on task-switching [30], performance tracking and performance-contingent rewards may be prime candidates for enhancing goal-directed behavioral control. Thus, we propose that boosting motivation via performance-contingent feedback (e.g., intrinsic and extrinsic rewards that promote task performance improvements) may serve as a useful tool in restoring flexibility in otherwise rigid behaviors.

Our Go/NoGo approach here primarily fills two major gaps that have limited the field: 1) The need for tools that capture existing associations that do not require experimental training or pre-training. If cue-response-outcome associations (e.g, green light–Go response) are so ingrained that they are relatively less flexible compared to newly learned associations (a purple–Go contingency), we can conclude that these familiar stimuli elicit habitual control—button press responses that are impervious to changes in task instructions. 2) The need for tools that allow the examination of habit disruption. The traditional tools are often ill-equipped to study habit disruption, because the habits that are examined are the product of the experiment. Because these habit-like behaviors are often not tied to an individual’s experiences outside of the lab, they are by definition newly-learned associations, and breaking these habits is trivial. To circumvent such limitations, our approach uses cues that are tied to specific meanings that can be adapted into a Go/NoGo task. Our test of habitual control involves manipulating the cue-response-outcome associations to determine whether participants persist in well-established behaviors that have been experienced outside of the lab, despite task instructions that are incongruent with these daily experiences. Thus, we are also able to investigate pre-test interventions (e.g., feedback) that may be able to protect individuals from inflexible responding.

The overall premise of the habit test is that goal-directed behaviors would be indicated by flexible responding, such that the goal-directed individual would produce comparable task performance regardless of color–response mapping necessary for obtaining the correct outcome. The habitual individual would respond in a manner that indicates that the associations congruent with daily experiences would prevail regardless of color–response mapping (i.e., significantly lower accuracy when color–response mappings are incongruent with thei real-world meanings). For example, despite the “correct” outcome requiring responses to the red light, the habitual individual may have a stronger go–press–correct association, negatively impacting performance. Such patterns would suggest that although changes to novel stimuli produce no difference in task performance to obtain the correct outcome, changes to familiar stimuli with real-world meanings significantly impact performance, biasing cue-based responding, possibly due to less-developed cue-response-outcome representations of the incongruent associations.

To achieve the goal of demonstrating and breaking a well-established habit, we introduce in Experiment 1 our novel Go/NoGo task that capitalizes on the familiar Green–Go, Red–NoGo associations people typically develop throughout the course of their lives. If the red–stop and green–go associations are well-learned, outcome-insensitive habits, there should be within-subject decrements in performance on an incongruent mapping of color to response (green–stop, red–go) compared to the well-learned congruent mapping (red–stop, green–go). That is, if participants are responding habitually, they should be more accurate when withholding responses to the red NoGo cue compared to the green NoGo cue (e.g., a within-subject difference in errors of commission across the congruent and incongruent associations). Importantly, such incongruency-related accuracy patterns should prevail regardless of the order in which these mappings are managed (i.e., performing the congruent contingencies first or second should not impact the results). In comparison, there should be no such within-subject differences between novel color–response mappings (e.g. blue–stop, purple–go vs. purple–stop, blue–go). Then, in Experiments 2 and 3, we explore strategies to disrupt the well-learned red–stop, green–go associations by amplifying the salience of the action outcomes. Specifically, we use cumulative performance-contingent feedback (Experiment 2), and performance feedback paired with a monetary incentive (Experiment 3) to attempt to reduce outcome-insensitive responses in the face of habit-eliciting stimuli.

## Experiment 1

### Methods

#### Participants

We recruited 50 undergraduate students (32 female, 18 male participants; *M*_Age_ = 20.28, *SD*_Age_ = 2.96) from the Rutgers University-Newark campus for course credit. All subjects provided written informed consent. Study protocols were approved by the Rutgers University Institutional Review Board. Participants were excluded if they reported having color-blindness.

#### Materials and procedures

**Fig 1:**
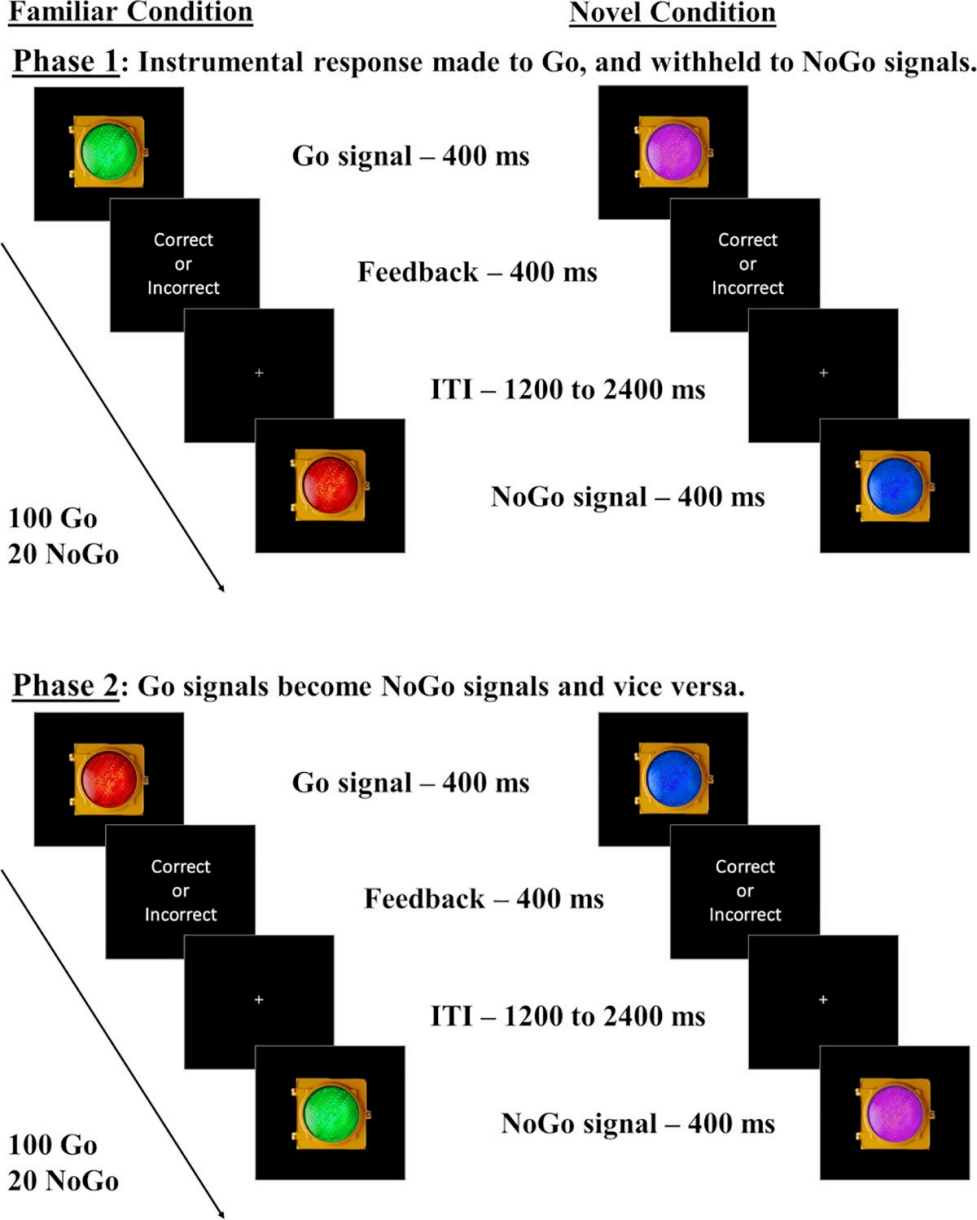
Go/NoGo task with familiar and novel lights. Participants are assigned to Familiar or Novel conditions. In the Familiar condition, subjects complete two phases: one where green signals Go and red signals NoGo (“congruent” mapping) and one where red signals Go and green signals NoGo (“incongruent” mapping). In the Novel condition, participants complete two similar phases, but the colors are blue and purple, for which there should be no strong pre-existing associations with “stop” and “go” responses. We predicted more commission errors in the Familiar condition for incongruent than congruent mappings, indicating outcome insensitivity, with no such within-subject differences expected in the Novel condition. Mapping orders were counterbalanced across subjects.

Participants were administered the Barratt Impulsivity Scale (BIS) [31], and randomly assigned to one of two stimulus familiarity (referred to as Stim_Familiarity hereafter) conditions, in that participants completed the task by managing either the Familiar (n = 25) or the Novel (n = 25) stimuli. They underwent a Go/NoGo task in which either green and red (Familiar condition) or purple and blue (Novel condition) traffic lights comprised Go and NoGo signals. Participants were instructed to respond as quickly and accurately to these stimuli as possible using the keyboard. A second phase followed in which the color–response contingencies were swapped (see Fig 1). Note that in the Familiar Stim_Familiarity condition, the green–Go/red–NoGo mapping was considered “congruent” with associations in everyday life, while the red–Go/green–NoGo mapping was considered “incongruent.” We assumed that the Novel stimuli have no well-established Go or NoGo associations in daily life. The Novel Stim_Familiarity condition serves the important role of a control group: if familiar associations are rigid, in that switching between red and green as the NoGo signal produces a decrement in within-subject accuracy, this change is only meaningful if switching between blue and purple Go/NoGo associations do not produce a within-subject decrease in performance. The order in which participants underwent the two phases of the task was counterbalanced to ensure that the results could not be attributed to a specific order of managing the contingencies. Thus, we were able to examine the rigidity of our Familiar behavioral contingencies hypothesized to elicit outcome-insensitive responses in relation to a Novel stimulus set. An exit survey with demographic information concluded the study.

Each phase comprised 100 Go and 20 NoGo trials (5:1 Go-NoGo ratio). The Go/NoGo stimuli remained onscreen for 400 milliseconds (ms), and each response produced a brief “correct” or “incorrect” text slide that offset after 400 ms (e.g., failure to withhold response in a NoGo trial produced the “incorrect” text slide). We reasoned that although trial-by-trial “correct/incorrect” feedback may protect goal-directed control by repeatedly displaying the outcome tied to the response (i.e., strengthening the response-outcome association), it serves an important purpose as an overt outcome event, similar to those present in traditional habit studies with stimulus–response–outcome associations [1,5,11,18,32]. Go responses had to be performed before stimulus offset to be registered as correct by pressing the “1” key on the keyboard. Late responses were treated as incorrect. The inter-trial intervals varied randomly between 1200 and 2400 ms to ensure engagement with the task. All participants completed a brief practice session (six correct Go or NoGo responses) using the same stimuli as the first phase. This practice session was conducted with the experimenter present to ensure the comprehension of instructions. The experimenter did not remain in the room while the participant completed the remainder of the task.

If these familiar associations elicit habitual, cue-driven behavioral control, subjects in the Familiar condition should experience a significant improvement in NoGo accuracy when congruent with lifelong experiences (red–NoGo), compared to when they are incongruent with lifelong experiences (green–NoGo). In contrast, because the blue and purple stimuli are not expected to have strong Go or NoGo associations, participants in the Novel condition should show similar performance levels for both novel color–response mappings (e.g., blue–NoGo vs. purple–NoGo), illustrating the flexibility of responses executed towards the novel stimuli.

#### Data analysis

Because the moderate ratio of Go to NoGo signals was expected to produce pre-potent Go responses [33], NoGo accuracy served as the primary measure of interest. As a secondary measure of outcome-sensitivity, identical analyses were performed using Go accuracy as dependent variable (DV). Go accuracy has been used as an index of attention [34,35]; here, we used Go accuracy to examine whether attention is recruited differently across familiar and novel associations (e.g., familiarity may recruit attentional processes to aid in executing color–response associations when these mappings are congruent with daily experiences, such as increased green–Go performance compared to red–Go).

We performed a mixed-design ANOVA with a DV of NoGo accuracy, Stim_Familiarity (Familiar or Novel stimulus conditions) as a between-subjects factor, and Color–Response_Mapping (congruent red–NoGo or incongruent green–NoGo mapping in the Familiar, and blue–NoGo vs. purple–NoGo color–response mapping in the Novel condition) as a within-subjects factor. We compared Age and Gender across Stim_Familiarity conditions to ensure that our results could not be attributed to arbitrary differences in demographic variables between the two groups. We also compared BIS Impulsivity scores across groups because of our interest in determining whether the stimuli in our task elicit habitual control regardless of a trait measure that may introduce individual differences in prepotent responses. None of these potential confounds were significantly different across groups (all *p*s> .05); therefore we performed the mixed-design ANOVA described above without covariates. Post-hoc t-tests were employed to detect mapping-related differences in both conditions when mixed-design ANOVAs yielded significant interactions.

We supplemented our analyses with a confirmatory omnibus test containing information from both conditions—a hierarchical multiple regression to test the predictive strength of the Stim_Familiarity variable on mapping-related change (i.e., NoGo accuracy difference across color–response mappings as DV). In short, the omnibus test closely follows the mixed-design ANOVAs and extracts R^2^ information from all controlled variables (regardless of whether group differences are evident across groups), an “Order” variable that codes for the counterbalanced order of Color–Response_Mappings, and the variables of interest, clearly displaying how much influence, if any, each set of regressors have on the DV. We summarize the omnibus regression data throughout the main text, but refer readers to the supplement for details. Similar analyses were performed with Go response time (RT) as DV to further explore the data. In addition to traditional null hypothesis testing methods, we also performed Jeffreys-Zellner-Siow (JZS) Bayes factor ANOVAs using JASP (version 0.11) that closely followed the primary mixed-design ANOVAs reported throughout [36,37]. We used the JZS method as it is suggested to be more conservative than the Bayesian information criteria method of deriving Bayes factors, and more suitable for mixed-design ANOVAs [38]. We derived Bayes factors (BF_10_, as in the evidence for the alternative hypothesis, H_1_, over the null hypothesis, H_0_) for Stim_Familiarity and Color–Response_Mapping main effects and their interaction to determine how much evidence our data provided in support of the Stim_Familiarity x Color–Response_Mapping interaction model for each DV over the null hypothesis and models that solely included the main effects. Bayesian methods and the associated statistical model tables corresponding to each analysis can be found in the Supplement.

To determine the sample size for our study, we performed an *a priori* power analysis using pilot data. We administered the task to 6 individuals (n = 3 per Stim_Familiarity condition), and using the pilot data effect size of the primary analysis of interest (Stim_Familiarity x Color–Response_Mapping interaction; Cohen’s *d* = 1.24), estimated that 12 participants would be needed per Stim_Familiarity condition to reach 80% statistical power. We doubled this sample size estimate to account for the Order factor nested into each Stim_Familiarity condition (the counterbalanced order in which participants were presented the Color–Response_Mappings in Familiar and Novel conditions), and recruited 50 participants.

### Results

#### Primary index of outcome-sensitivity: NoGo accuracy

**Fig 2:**
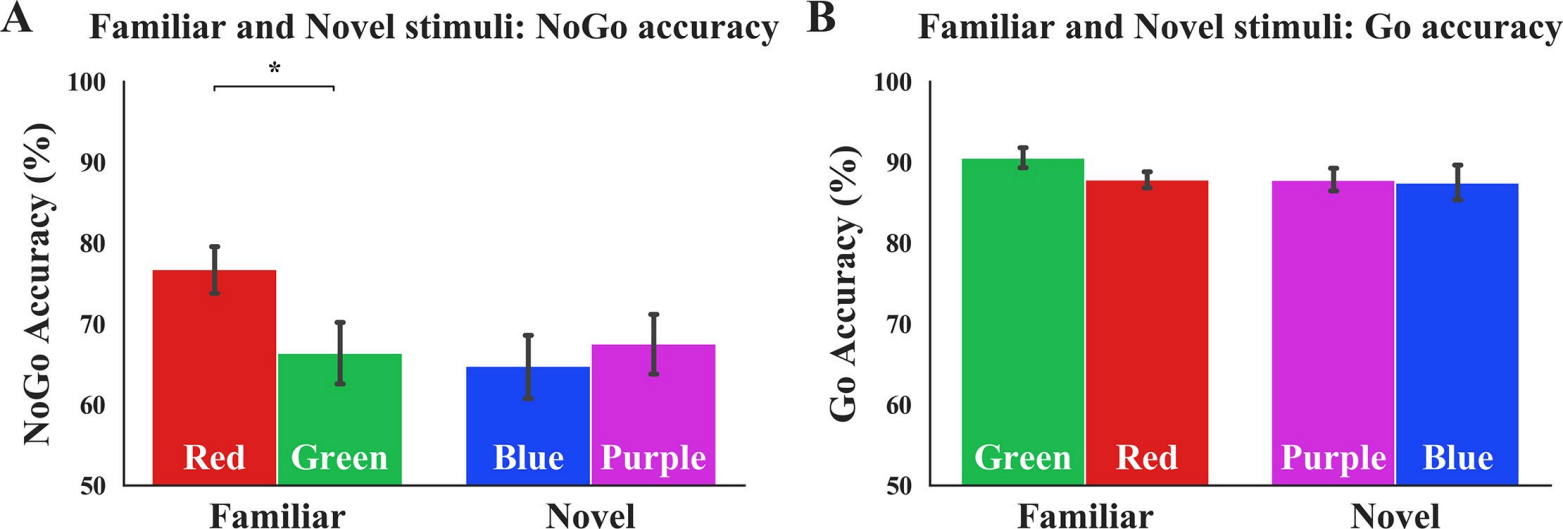
NoGo and Go performance to familiar and novel stimuli. **(A)**. Familiar stimuli elicit mapping-related change in NoGo accuracy. Participants make significantly fewer errors of commission when the NoGo signal is red compared to green. There is no difference in accuracy in the Novel condition when the NoGo signal is purple vs. blue. Stim_Familiarity x Color–Response_Mapping interaction: *p* = .004. **(B)** Familiar stimuli do not significantly elicit mapping-related change in Go accuracy. There is no significant difference in Go accuracy when Go signal is red compared to green. Likewise, no differences are seen in the Novel condition, when Go signal is blue vs. purple. Stim_Familiarity x Color–Response_Mapping interaction: *p* = .140. Error bars depict standard error of mean (SEM). Color of bars reflects NoGo and Go stimulus colors.

To examine whether Stim_Familiarity (Familiar or Novel) predicted outcome-sensitivity, we performed a repeated measures ANOVA using NoGo accuracy as the DV, Stim_Familiarity as a between-subjects factor, Color–Response_Mapping as a within-subjects factor. We found no main effect of Stim_Familiarity, *F*(1,48) = 1.36, *p* = .249, η_p_^2^ = .03, or Color–Response_Mapping, *F*(1,48) = 3.08, *p* = .086, η_p_^2^ = .06. but as evident in Fig 2A, we found a significant Stim_Familiarity x Color–Response_Mapping interaction. *F*(1,48) = 9.29, *p* = .004, η_p_^2^ = .16. The congruent mapping produced higher accuracy compared to the incongruent mapping, which was not different from performance to the novel stimuli. Post-hoc paired-samples t-tests further revealed a significant difference in NoGo accuracy in the Familiar condition, *t*(24) = 3.53, *p* = .002, suggesting that the congruent “red–NoGo” mapping elicits fewer errors of commission compared to the incongruent “green–NoGo” mapping—a difference indicative of outcome-insensitive, habitual control. Contingency change yielded no differences in errors of commission between phases in the Novel condition, supporting the labile nature of newly learned associations, *t*(24) = -0.88, *p* = .387.

The omnibus regression test confirmed the significant effect of Stim_Familiarity. When controlling for participants’ Age, Gender, and self-reported Impulsivity, and accounting for the Order variable that codes for order of Color–Response_Mapping presentation, the inclusion of the Stim_Familiarity regressor in the hierarchical multiple regression model explained an additional 15.5% of the variance in outcome-sensitivity: β_Stim_Familiarity_ = -0.40, *p* = .006, ΔR^2^ = .15, indicating differential outcome-sensitivity across Familiar and Novel conditions. The details of this omnibus regression test and beta weights of all model parameters can be found in the supplement (S2 Table in S1 File).

#### Secondary index of outcome-sensitivity: Go accuracy

To further examine the potential effect of color–response familiarity on outcome-sensitivity via attentional processing, we performed a mixed-design ANOVA using Go accuracy as the DV. This analysis revealed no main effect of Stim_Familiarity, *F*(1,48) = 0.57, *p* = .452, η_p_^2^ = .01, a near-significant main effect of Color–Response_Mapping, *F*(1,48) = 3.84, *p* = 0.056, η_p_^2^ = .07, and no Stim_Familiarity x Color–Response_Mapping interaction, *F*(1,48) = 2.26, *p* = .140, η_p_^2^ = .04 (Fig 2B).

The omnibus regression test revealed a significant role played by Stim_Familiarity on our secondary assay of outcome-sensitivity, Go accuracy. Controlling for participants’ Age, Gender, and Impulsivity scores, and accounting for the Order variable that codes for order of Color–Response_Mapping presentation, the inclusion of the Stim_Familiarity regressor significantly predicted mapping-related Go accuracy changes: β_Stim_Familiarity_ = -0.27, ΔR^2^ = .07, *p* = .049 (see S4 Table in S1 File). The discrepancy in the omnibus regression and the mixed-design ANOVA results is potentially due to the inclusion of Age, Gender, and Impulsivity as controlled regressors in the regression. Lastly, similar analyses performed with Go RT as DV yielded no significant results (all *p*s > .05).

### Discussion

This experiment demonstrates that habitual behavior that capitalizes on existing, non-lab-derived associations, can be demonstrated in the lab. By using the strong links between the green–go and red–stop associations in a Go/NoGo task, we were able to quantify the degree of flexibility to well-stamped in cue–response–outcome associations. Importantly, our results suggest that responses are more outcome-insensitive (i.e., habitual) when the stimulus meanings are congruent with our experiences in daily life (e.g., when a traffic light indicating “stop” is red, rather than green, blue or purple). This finding is in line with a previous report of improved inhibitory performance when the stop-signal is red compared to green [22], which provides further support to the notion that an environmentally congruent cue can substantially affect action execution. However, the incongruency-related changes alone are not enough to conclude that a response is habitual; rather this conclusion must be verified by a comparison of the habitual associations (i.e., green–go, red–stop) with the novel control condition Go/NoGo associations (i.e., purple–go, blue–stop). Specifically, these red and green light stimuli triggered outcome-insensitive actions as evidenced by an accuracy change when Go and NoGo contingencies were incongruent with their well-established meanings outside of the lab. In contrast, the novel purple–go and blue–stop contingencies are not well-established in one’s daily experiences, and their associative strength is limited to the participant’s brief experience in the lab. Therefore, compared to the familiar stimuli, the actions evoked by the novel stimuli are more flexible to contingency changes, as reflected by similar NoGo and Go accuracy scores for blue vs. purple. Our Go accuracy omnibus regression results allude to the notion that while red and green stimuli are rigid and habitual in triggering stop/go actions, blue and purple stimuli are not strongly associated with either of the stop/go outcomes, but instead are labile and sensitive to the changes in action–outcome contingencies. Possibly, because Go accuracy has been used as an index of attention [34,35], these results may be due to heightened recruitment of attentional processing when contingencies are familiar and congruent with daily experiences. However, the Go accuracy results are sensitive to the inclusion of controlled covariates (which we had elected to exclude from the primary mixed-design ANOVA due to the lack of differences between groups). Thus, we interpret these Go accuracy results with caution, and suggest that replications in further examinations are warranted to better understand the attentional underpinnings in well-learned versus novel associations.

It can be argued that if these familiar red and green stimuli elicit outcome-insensitive habits, one should also display lower accuracy rates to green–NoGo compared to blue or purple–NoGo contingencies. However, our results above suggest that green–NoGo performance is similar to those elicited by the novel stimuli. The comparable performance here may be due to Type II error. We based our sample size estimates on the primary Stim_Familiarity x Color–Response_Mapping interaction of interest, which essentially compares the difference between red and green NoGo performance to the difference between blue and purple NoGo performance. Possibly, the nuances in the data such as the specific differences between green and blue/purple NoGo performance may require more power, thus making it more difficult to detect a potential decrement in green–NoGo accuracy with n = 50. We further examine the unexpected pattern observed here in Experiments 2 and 3, with the prediction that with an increased sample size, the green–NoGo mapping will indeed elicit lower accuracy rates compared to either Novel condition NoGo mapping.

## Experiment 2

In Experiment 2, we attempt the breaking of well-learning habits by boosting motivation via cumulative performance feedback prior to contingency reversal. Because the motivational control framework attributes habits to be driven by antecedent cues and goal-directed actions to be guided by resulting outcomes [1], we hypothesized that amplifying the salience of the outcome may promote goal-directed performance at the expense of habitual control, thus aiding in breaking the well-learned habit.

### Methods

#### Participants

We recruited 100 undergraduate students (67 female and 33 male participants; *M*_Age_ = 20.26, *SD*_Age_ = 3.05) from the Rutgers University-Newark campus. All participants provided written informed consent and received course credit for their participation. Study protocols were approved by the Rutgers University Institutional Review Board. Participants were excluded if they reported having color-blindness.

#### Procedures

For the Go/NoGo task, participants were randomly assigned to a Feedback Group (n = 50) or No-Feedback Group (n = 50). Within each group, participants were further divided, with 25 participants in each group managing Familiar, and 25 participants managing Novel (Stim_Familiarity conditions) stimuli (total n = 100).

Feedback Group: After completing the BIS, participants underwent a similar Go/NoGo task to the one described in Experiment 1. Accordingly, each phase comprised 100 Go and 20 NoGo trials (5:1 Go–NoGo ratio). As reported in Experiment 1, all stimuli remained on the screen for 400 ms, and responses produced brief feedback slides consisting of “correct” or “incorrect” that offset after 400 ms (e.g., failure to withhold response in a NoGo trial produced the “incorrect” text slide). Go responses had to be performed before stimulus offset to be registered as correct by pressing the “1” key on the keyboard. Late responses were treated as incorrect. The inter-trial intervals varied randomly between 1200 and 2400 ms to ensure engagement with the task. All subjects completed a brief practice session (six correct Go or NoGo responses) using the same stimuli that comprised the task. This practice session was conducted with the experimenter present to ensure the comprehension of instructions.

In the Familiar condition, participants were instructed to “Go” on green traffic light stimuli as quickly and accurately as possible, and withhold responses to the red traffic light. Next, a cumulative performance feedback manipulation followed, in which we displayed participants’ cumulative NoGo accuracy as a percentage score on the screen. Specifically, upon completion of the first half of the task, the experimenter re-entered the room, and displayed to the participant a percentage score, instructing that it reflected their performance thus far (they were not informed that the score only reflected NoGo accuracy). Participants were further instructed that in the next phase of the task, the Go and NoGo signals would be reversed, such that they would need to make a response as quickly and accurately as possible to the red traffic light, and refrain from responding to the green traffic light. Identical feedback and task instructions were provided to the participants in the Novel condition regarding the change in contingencies of the purple–Go and blue–NoGo associations. It should be noted that Experiment 1 reports differential mapping-related change across Familiar and Novel conditions regardless of the order in which phases were completed (S2 and S4 Tables in S1 File). Unlike Experiment 1, the phase orders in Experiment 2 were not counterbalanced, in that all participants in the Familiar condition underwent the congruent (green–Go, red–NoGo) mappings first, followed by the incongruent mappings; all participants in the Novel condition underwent the purple–Go, blue–NoGo mapping first, and these mappings were reversed in the second phase. This change in experimental protocol enabled rendering the congruent contingency as baseline for participants in the Familiar group, and testing whether the presence of a mid-experiment performance manipulation affected subsequent incongruent task performance. An exit survey consisting of demographic questions concluded the experiment.

No-Feedback Group: Participants in the No-Feedback group underwent the same procedures as the Feedback group, except that no cumulative performance feedback was provided at any point. Specifically, the experimenter re-entered the room upon completion of the first half of the task, but did not display any performance information to the participant, and instead proceeded with task instructions. This No-Feedback group served as a control condition for the Feedback group, as well as an internal replication of Experiment 1.

#### Data analysis

To replicate our primary Experiment 1 finding of mapping-related accuracy change exclusively in the Familiar Stim_Familiarity condition, we performed a mixed-design ANOVA using data from the No-Feedback group, with Stim_Familiarity as the between-subject and Color–Response_Mapping as the within-subject factor, with NoGo accuracy as DV. Neither Age, Gender, nor Impulsivity significantly differed across Stim_Familiarity groups (all *p*s > .05), and thus were not included in the mixed-design ANOVAs.

To examine the role of Feedback, we performed mixed-design ANOVAs with NoGo accuracy as DV, Feedback as a between-subjects and Color–Response_Mapping as a within-subjects factor for each Stim_Familiarity condition. Post-hoc t-tests were carried out to examine mapping-related accuracy differences in both Feedback groups following significant Feedback x Color–Response_Mapping interactions. Similar to the analysis above, Age, Gender, and Impulsivity were not significantly different across Feedback groups in the Familiar Stim_Familiarity condition (all *p*s> .05), therefore we performed the mixed-design ANOVAs without covariates. In the Novel Stim_Familiarity condition, Age significantly differed across Feedback groups, Levene’s corrected *t*(33.48) = 2.24, *p* = .032, and thus Age was included as a covariate in the mixed-design ANOVA that examined Feedback differences when managing Novel associations. Neither Gender nor Impulsivity differed across Feedback groups in the Novel Stim_Familiarity condition (both *p*s > .05).

As a secondary measure of outcome-sensitivity via attentional processing, identical analyses were performed using Go accuracy as a DV. Similar analyses were performed with Go RT as DV to further explore the data. It should be noted that we did not test for a three-way Stim_Familiarity x Feedback x Color–Response_Mapping interaction with any of our DVs, because our primary interest was determining whether cumulative performance feedback has any effect on outcome-sensitivity, not necessarily whether this effect differs based on the familiarity of the stimuli. For example, we would not expect cumulative feedback to promote accuracy improvements in the Familiar Stim_Familiarity while impairing performance in the Novel condition.

Building on Experiment 1, we followed up on each mixed-design ANOVA with Bayesian statistics, and performed a confirmatory omnibus hierarchical multiple regression to test the predictive strength of the Stim_Familiarity and Feedback variables on mapping-related change. Matching Bayesian analyses of each mixed-design ANOVA can be found in the Supplement (S5 and S6 Tables in S1 File for the replication of Experiment 1; S7, S8, S10, and S11 Tables in S1 File for cumulative performance feedback-related Bayesian analyses). The summary of the omnibus regression test is reported below, and its details can be found in the supplement (S9 and S12 Tables in S1 File).

We doubled our Experiment 1 sample size of n = 50 to n = 100 to ground our feedback-related hypotheses on a replicated effect of habitual performance to familiar, and goal-directed performance to novel stimuli, while also accounting for the Feedback and No-Feedback groups that are nested into the Familiar and Novel Stim_Familiarity conditions.

### Results

#### Replication of Experiment 1 results

Focusing specifically on the No-Feedback group, we performed a mixed-design ANOVA using Stim_Familiarity as the between-subjects factor, Color–Response_Mapping as the within-subjects factor, and NoGo accuracy as DV. We found no main effect of Stim_Familiarity, *F*(1,48) < 0.01, *p* = .942, η_p_^2^ < .01, a main effect of Color–Response_Mapping, *F*(1,48) = 9.16, *p* = .004, η_p_^2^ = .16, and a near-significant Stim_Familiarity x Color–Response_Mapping interaction, *F*(1,48) = 3.77, *p* = .058, η_p_^2^ = .07.

We performed the same analysis using Go accuracy as DV, and found no main effect of Stim_Familiarity, *F*(1,48) = 0.63, *p* = .433, a main effect of Color–Response_Mapping, *F*(1,48) = 8.30, *p* = .006, and a significant Stim_Familiarity x Color–Response_Mapping interaction, *F*(1,48) = 6.39, *p* = 0.015. Post-hoc paired-samples t-tests on this significant interaction show that Go accuracy was significantly lower when incongruent with real-world mappings in the Familiar Stim_Familiarity condition, *t*(24) = 3.10, *p* = .005, whereas both mappings in the Novel Stim_Familiarity condition produced similar Go accuracy rates, *t*(24) = 0.28, *p* = .785. These results suggest that our Experiment 1 findings are supported on a separate sample of participants in Experiment 2 (while also revealing the expected Go accuracy effect not detected in Experiment 1), although the near-significant NoGo accuracy effect (*p* = .058) warrants further interrogation in Experiment 3.

#### Primary index of outcome-sensitivity: NoGo accuracy

**Fig 3:**
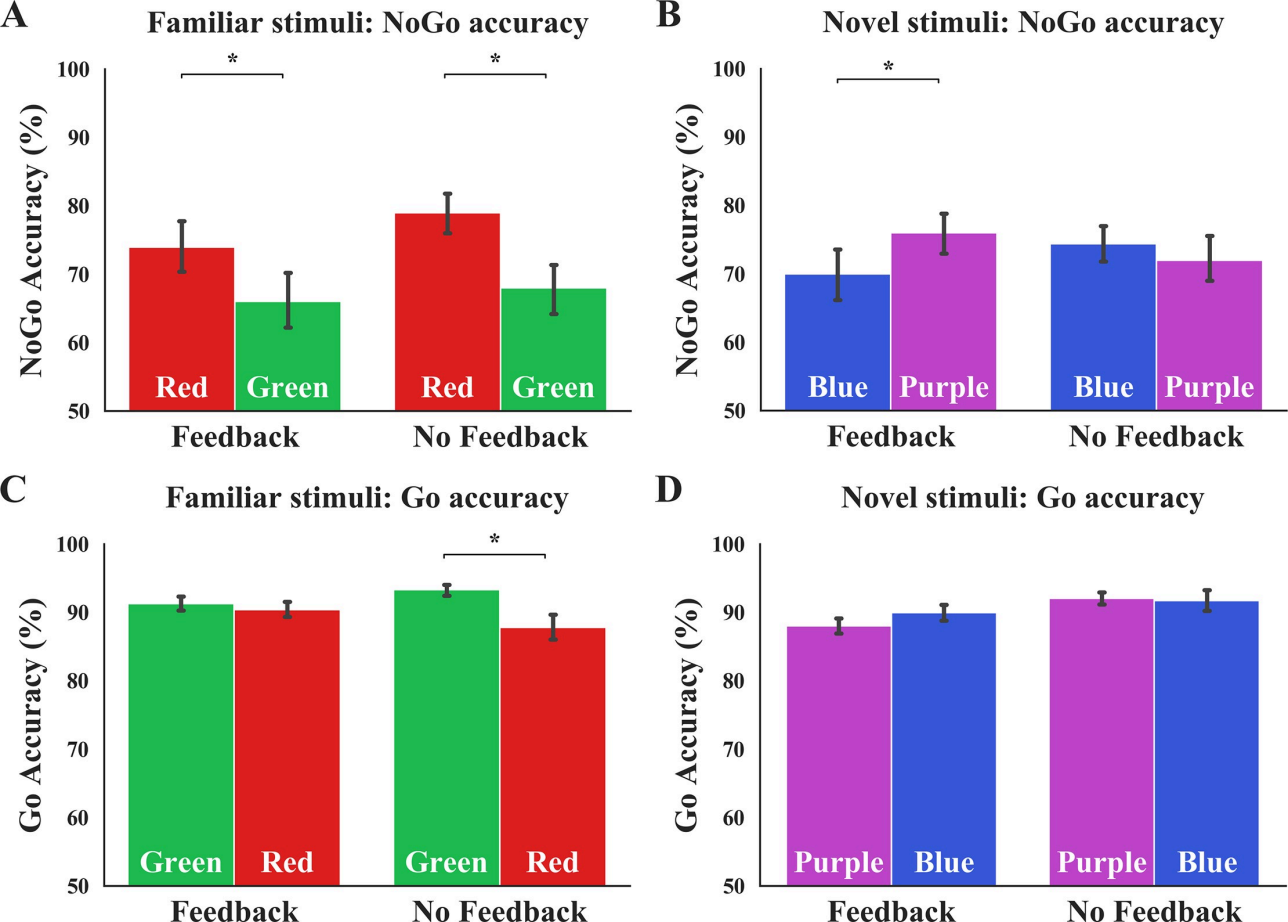
The effects of performance feedback on NoGo and Go accuracy. **(A)** Performance feedback does not significantly disrupt well-established habits. In the Familiar condition, both Feedback and No-Feedback groups display a similar mapping-related change (interaction *p* = .513) in NoGo accuracy. Note that the non-significant interaction indicates the comparable mapping-related accuracy change across feedback groups. **(B)** NoGo accuracy in the Novel condition is significantly improved by performance feedback (sig. interaction of *p* = .028) when controlling for Age (which was significantly different across the Feedback groups). **(C)** Performance feedback protects against habitual Go actions. Providing cumulative performance feedback prevented the mapping-related Go accuracy change when managing Familiar stimuli (Feedback x Color–Response_Mapping interaction *p* = .019). **(D)** Performance feedback did not significantly improve Go accuracy in the Novel condition (Feedback x Color–Response_Mapping interaction *p* = .05). Error bars denote SEM. Color of bars reflects NoGo and Go stimulus colors.

Familiar condition: We hypothesized that performance feedback may be a salient factor that can potentially restore goal-directed control when managing these well-established associations. However, cumulative performance feedback did not break the habits elicited by these familiar stimuli. We performed a mixed-design ANOVA using NoGo accuracy as the DV, Feedback as the between-subjects, and Color–Response_Mapping as the within-subjects factor. We found no main effect of Feedback, *F*(1,48) = 0.53, *p* = .469, η_p_^2^ = .01, a main effect of Color–Response_Mapping, *F*(1,48) = 17.40, *p* < .001, η_p_^2^ = .27, but no significant Feedback x Color–Response_Mapping interaction: *F*(1,48) = 0.43, *p* = .513, η_p_^2^ = .01 (see Fig 3A). Overall, these results indicate that cumulative performance feedback did not prevent habitual control from dominating in the Familiar condition, as accuracy in both Feedback groups were significantly changed when contingencies were incongruent with daily experiences (as evident by the main effect of Color–Response_Mapping, but no Feedback x Color–Response_Mapping interaction; see S9 Table in S1 File for supporting omnibus regression results). Although we were unable to break habits as hypothesized here, our findings lend support to the rigidity of these well-learned associations that persevere in the face of performance feedback, which is theorized to be an otherwise salient motivational manipulation [39,40].

Novel condition: We performed a similar ANOVA to determine whether cumulative performance tracking improved goal-directed control of novel associations. We included Age as a covariate, as this variable was significantly different across Feedback groups in the Novel Stim_Familiarity condition, Levene’s corrected *t*(33.48) = 2.24, *p* = .032. As seen in Fig 3B, we did not find a main effect of Feedback, *F*(1,47) = 0.32, *p* = .573, η_p_^2^ = .01, found a near-significant main effect of Color–Response_Mapping, *F*(1,47) = 3.05, *p* = .087, η_p_^2^ = .06, and found a Feedback x Color–Response_Mapping interaction on NoGo accuracy in the Novel Stim_Familiarity when controlling for Age: *F*(1,47) = 5.16, *p* = .028, η_p_^2^ = .10. Post-hoc t-tests probing this interaction indicated no significant differences related to changes in mapping in the No-Feedback group, *t*(24) = 0.88, *p* = .390, and a non-significant improvement following cumulative performance feedback in the Feedback group, *t*(24) = 1.62, *p* = .118. The discrepancy in the significant interaction and non-significant t-test may be due to the inclusion of the Age covariate in the ANOVA, whereas the t-tests do not control for any variables (see S9 Table in S1 File in the Supplement for supporting omnibus regression reports). In sum, these results suggest that despite the slight improvement observed in the Novel Stim_Familiarity condition, cumulative performance feedback alone may not be a salient enough manipulation to restore goal-directed control.

#### Secondary index of outcome-sensitivity: Go accuracy

Familiar condition: We performed a mixed-design ANOVA of the Familiar condition data using Go accuracy as DV, Feedback as a between-, and Color–Response_Mapping as a within-subjects factor. We found no significant main effect of Feedback *F*(1,48) = 0.03, *p* = .852, η_p_^2^ < .01, a main effect of Color–Response_Mapping, *F*(1,48) = 11.57, *p* < .001, η_p_^2^ = .19, and found a significant Feedback x Color–Response_Mapping interaction: *F*(1,48) = 5.90, *p* = .019, η_p_^2^ = .11 (Fig 3C), suggesting that Go accuracy was affected differentially by performance feedback, possibly by performance feedback promoting the recruitment of attentional processing to Go stimuli. Post-hoc paired-samples t-tests of Go accuracy across phases yielded evidence for an incongruency-related change in the No-Feedback group, *t*(24) = 3.22, *p* = .004), but not in the Feedback group, *t*(24) = 1.14, *p* = .265. Furthermore, the Stim_Familiarity and Feedback regressors are significant in predicting Go accuracy change in the omnibus hierarchical regression (β_Stim_Familiarity_ = -0.32, *p* = .001, β_Feedback_ = 0.28, *p* = .003; ΔR^2^ = .18; see S12 Table in S1 File). These patterns suggest that although we did not see a significant habit disruption effect in our primary NoGo accuracy DV, performance feedback may be adaptively reallocating attention when the task entails overriding a well-established green–Go association.

Novel condition: Despite the significant Feedback regressor in the omnibus test, we did not observe a significant improvement effect due to cumulative performance feedback in the Novel condition Go accuracy results. A mixed-design ANOVA using Go accuracy as the DV, Feedback as the between-, and Color–Response_Mapping as the within-subjects factor, with Age, as covariate (due to the significant Age differences across Feedback groups in this Novel Stim_Familiarity condition) revealed no significant main effect of Feedback, *F*(1,47) = 2.24, *p* = .141, η_p_^2^ = .05, or Color–Response_Mapping, *F*(1,47) = 2.48, *p* = .140, η_p_^2^ = .05, and a near-significant Feedback x Color–Response_Mapping interaction: *F*(1,47) = 4.03, *p* = .05, η_p_^2^ = .08 (Fig 3D). Given the lack of significant Feedback x Color–Response_Mapping interaction in the Novel condition (*p* = .05), we refrain from speculating further about the effect of cumulative performance feedback on goal-directed Go responses. Similar analyses performed with Go RT as DV yielded no significant findings (all *p*s > .05).

### Discussion

Although the No-Feedback group successfully replicated the findings of Experiment 1, results from the Feedback group indicate that cumulative performance feedback is not sufficient to disrupt the well-learned habits elicited by the familiar stimuli used in our task. However, supplementary analyses using accessory measures of attentional processing (i.e., familiar Go accuracy), suggest that feedback may be a useful tool in enhancing behavioral flexibility via the reallocation of attention. Therefore, these patterns warrant further examination of feedback to disrupt habitual control.

When interpreting the potential effects of cumulative performance feedback, it should be noted that in all versions of the task there also exist trial-by-trial performance indicators. Participants are provided immediate feedback following each response (or lack of response) in the form of a brief “correct” or “incorrect” slide. This immediate, trial-by-trial feedback event serves the important purpose of completing the stimulus–response association with an explicit outcome component (i.e., stimulus: go/nogo signal, response: button press or no press, outcome: trial-by-trial feedback), as these associations are often trained in the traditional habit task [1,5,11,18,32]. Possibly, the presence of trial-by-trial feedback may already protect goal-directed control by repeatedly strengthening the response–outcome association, thus limiting any additional effect that cumulative performance feedback may provide. With that said, given that trial-by-trial feedback is provided in all trials, color–response mappings, and feedback groups, our results are not confounded by this type of feedback; any potential effect of trial-by-trial feedback should be apparent across all participants.

We conclude that cumulative performance feedback was not salient enough to break habits according to our primary analyses, yet our findings were valuable in two ways. First, the validity of our Go/NoGo task using well-learned associations to study habits relies on the rigidity of these green–go and red–stop associations. The persistent habitual control exhibited here despite the delivery of performance feedback lends credence to the associative strength of our familiar stimuli. Next, given the modest signs of performance improvement due to the presentation of performance information, early reports of combined (i.e., performance tracking and monetary incentives) feedback’s positive effects on performance, and the beneficial effects of performance-contingent feedback on behavioral flexibility [25–29], we were motivated to enhance the salience of the provided feedback to break well-learned habits. In Experiment 3, we further amplified the salience of the outcome by pairing performance-contingent cumulative feedback with a bonus monetary reward prior to changing Go and NoGo contingencies. We studied the effects of monetary and cumulative performance feedback on Go/NoGo task performance, and whether this amplification of outcome salience resulted in the breaking of a well-learned habit, and improvement of novel, goal-directed performance.

## Experiment 3

The promising but insufficient effect of cumulative performance feedback on the motivational control of action motivated us to examine the combined effect of performance and monetary input. Thus, we implemented in our mid-experiment performance feedback manipulation a cash bonus. We hypothesized that this bonus, delivered by the experimenter, and combined with performance tracking information, would enhance goal salience and promote cognitive control processes to override habitual control. Experimental procedures were identical to those described in Experiment 2, with the addition of awarding participants in the Feedback group a surprise $5 cash bonus before the change in Go/NoGo mappings.

### Methods

#### Participants

To test the effects of dual feedback, we recruited the same number of participants for Experiment 3 as in Experiment 2. One-hundred participants (76 female, 24 male participants; *M*_Age_ = 19.74, *SD*_Age_ = 2.79) from the Rutgers University-Newark undergraduate research subject pool were recruited for course credit. All participants provided written informed consent. Study protocols were approved by the Rutgers University Institutional Review Board. Participants were excluded if they reported having color-blindness.

#### Procedures

After completing BIS, participants underwent a similar Go/NoGo task to the one described in Experiment 2, where they were randomly assigned to Feedback and No-Feedback groups. Participants were further divided within each group, with 25 in each group managing the Familiar, and 25 managing Novel (Stim_Familiarity conditions) stimuli (total n = 100). As in Experiment 2, each phase comprised 100 Go and 20 NoGo trials (5:1 Go–NoGo ratio), and the stimuli remained on the screen for 400 ms. Go and NoGo responses (or lack thereof) produced brief feedback slides consisting of “correct” or “incorrect” that offset after 400 ms (e.g., failure to withhold response in a NoGo trial produced the “incorrect” text slide). Go responses had to be performed before stimulus offset to be registered as correct by pressing the “1” key on the keyboard. Late responses were treated as incorrect. The inter-trial intervals varied randomly between 1200 and 2400 ms to ensure engagement with the task. All participants completed a brief practice session prior to the task, similar to the previous two experiments.

Identical to Experiment 2, in the Familiar condition’s first phase, participants were instructed to “Go” on green traffic light stimuli as quickly and accurately as possible, and “NoGo” on red traffic light stimuli. Next, a monetary and cumulative performance feedback manipulation followed, in which we displayed participants’ cumulative NoGo accuracy as a percentage score on the screen. Upon completion of the first half of the task, the experimenter re-entered the room, and displayed to the participant a percentage score, instructing that it reflected their performance thus far. Additionally, unique to Experiment 3, the experimenter left the room following the performance feedback display, and returned briefly after with a $5 bill, informing the participant that this money was earned because of performance thus far in the task. Unbeknownst to the participants, the cash bonus was not actually contingent on performance, in that the $5 was provided to all participants in the Feedback group. However, the participants were indeed instructed that the cash bonus was paid because of their performance, linking the monetary incentive to task performance. The participant was then informed that the Go and NoGo signals would be reversed, such that they would need to make a response as quickly and accurately as possible to the red traffic light, and refrain from responding to the green traffic light. Identical performance and monetary feedback information and reversal instructions were provided to the participants in the Novel condition regarding the reversal of purple–Go and blue–NoGo responses. An exit survey containing demographic questions concluded the experiment.

Participants in the No-Feedback group underwent the same procedures as the Feedback group, except for the feedback manipulation, in that participants received no cumulative performance or monetary feedback. Specifically, all experimental procedures across groups were identical, except for the experimenter’s delivery of performance information and the handing of the cash bonus. The experimenter re-entered the room upon the participant’s completion of the first half of the experiment, briefly left the room, and returned again (similar to the Feedback group procedures), and started the participant on the second half of the study without the performance information or cash bonus.

#### Data analysis

To replicate our primary Experiment 1 finding of mapping-related accuracy change exclusively in the Familiar Stim_Familiarity condition, similar to Experiment 2, we performed a mixed-design ANOVA in the No-Feedback group with Stim_Familiarity as the between-subject and Color–Response_Mapping as the within-subject factor, with NoGo accuracy as DV. Neither Age nor Impulsivity significantly differed across Stim_Familiarity conditions (both *p*s > .05), yet Gender differed across conditions, *χ2* (1) = 4.37, *p* = .037; thus we only included Gender as a controlled variable in the mixed-design ANOVA. We repeated this analysis with Go accuracy as DV to replicate the corresponding analyses in Experiments 1 and 2.

To reveal the potential effect of dual feedback on outcome-sensitivity, we performed mixed-design ANOVAs with NoGo accuracy as the DV, Feedback as a between- and Color–Response_Mapping as a within-subjects factor for each Stim_Familiarity condition, using Gender as a covariate. Post-hoc paired-samples t-tests were carried out when necessary to examine mapping-related accuracy differences in both Feedback groups. As a supplemental measure of outcome-sensitivity, identical tests were performed using Go accuracy (i.e., a marker of attentional processing, [34,35]) as the DV. Similar analyses were performed with Go RT as DV to further explore the data.

Identical to Experiment 2, we followed up on each mixed-design ANOVA with Bayesian statistics, and performed a confirmatory omnibus hierarchical multiple regression to test the predictive strength of the Stim_Familiarity and Feedback variables on outcome-sensitivity. The results of each Bayesian analysis can be found in the Supplement (S13-S18 Tables in S1 File). The summary of the omnibus regression tests are reported below, and the details can be found in the Supplement (S19 and S20 Tables in S1 File). Lastly, in Experiment 1, we had not detected a specific impairment in green–NoGo accuracy compared to newly-learned NoGo associations (e.g., purple–NoGo). Our inability to detect this effect may be due to insufficient power. Experiment 1’s power analysis was based on pilot data from 6 individuals, and estimated the sample size needed to reach 80% power in detecting a Stim_Familiarity x Color–Response_Mapping interaction. Although the interaction is critical for demonstrating the expected habit expression effect, a larger sample size may be needed to explore the nuances in the data, such as specific comparisons of green–NoGo and blue/purple–NoGo performance. Thus, we pooled No-Feedback data from Experiments 2 and 3 to reach n = 100, and using an independent-samples t-test, further explored whether green–NoGo (i.e., the color–response mapping that is incongruent with daily experiences) elicits lower accuracy rates compared to purple–NoGo (i.e., the corresponding newly-learned color–response mapping). We chose to pool the No-Feedback groups in these experiments, as they are the most comparable with Experiment 1 in procedure, and chose to compare green and purple, as both of these colors served as reversed NoGo signals in Experiments 2 and 3.

### Results

#### Replication of Experiment 1 results

Similar to Experiment 2, focusing specifically on the No-Feedback group, we performed a mixed-design ANOVA using Stim_Familiarity as the between-subjects factor, Color–Response_Mapping as the within-subjects factor, and NoGo accuracy as DV to replicate our original Experiment 1 findings of well-learned habit expression. Gender was significantly different across the Stim_Familiarity conditions, and thus served as a covariate. We found no main effect of Stim_Familiarity, *F*(1,47) = 3.03, *p* = .088, η_p_^2^ = .06, a main effect of Color–Response_Mapping, *F*(1,47) = 13.33, *p* = .001, η_p_^2^ = .22, and a significant Stim_Familiarity x Color–Response_Mapping interaction, *F*(1,47) = 12.65, *p* = .001, η_p_^2^ = .21. Post-hoc paired-samples t-tests indicated that NoGo accuracy was significantly lower when incongruent with real-world mappings in the Familiar Stim_Familiarity condition, *t*(24) = 5.25, *p <* .001, Both Color–Response_Mappings in the Novel Stim_Familiarity condition produced comparable NoGo accuracy rates, *t*(24) = 0.08, *p* = .938. These results replicate the significant habit expression effects seen in Experiment 1 (and the near-significant effect in Experiment 2), bolstering the idea that habit expression is evident exclusively when managing Familiar stimuli.

We performed the same analysis using Go accuracy as DV, and found no main effect of Stim_Familiarity, *F*(1,47) = 0.10, *p* = .923, η_p_^2^ < .01, no main effect of Color–Response_Mapping, *F*(1,47) = 2.32, *p* = .134, η_p_^2^ = .05, and a significant Stim_Familiarity x Color–Response_Mapping interaction, *F*(1,47) = 5.25, *p* = .027, η_p_^2^ = .10. Post-hoc paired-samples t-tests on this significant interaction showed that Go accuracy was significantly lower when incongruent with daily experiences in the Familiar Stim_Familiarity condition, *t*(24) = 2.58, *p* = .017, whereas both mappings in the Novel Stim_Familiarity condition produced similar Go accuracy rates, *t*(24) = 0.51, *p* = .616. These results suggest that our Experiment 1 findings, which were replicated in Experiment 2, are further validated in Experiment 3 (while also revealing the expected Go accuracy effect not detected in Experiment 1).

#### Primary index of outcome-sensitivity: NoGo accuracy

**Fig 4:**
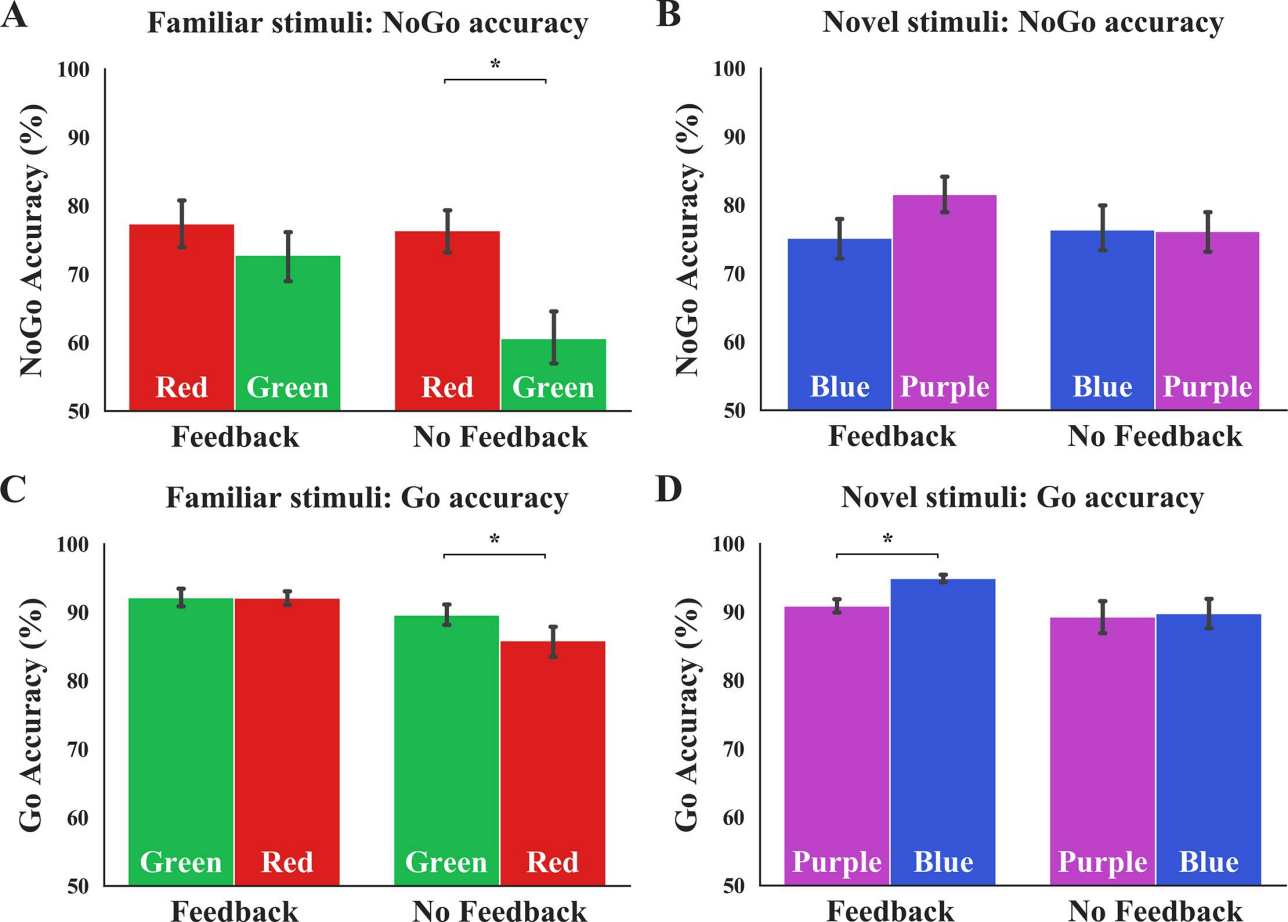
The effects of combined monetary and performance feedback on NoGo and Go accuracy. **(A)** Monetary and performance feedback disrupt habits while improving goal-directed performance to newly-learned stimuli. Providing performance and monetary feedback protects against the mapping-related change when overriding well-learned associations (Feedback x Color–Response_Mapping interaction: *p* = .005). **(B)** The effect of dual feedback on goal-directed control of novel associations fell short of significance (Feedback x Color–Response_Mapping interaction: *p* = .066, controlling for Gender). **(C)** Dual feedback improves goal-directed Go accuracy. Dual feedback had a significant effect on mapping-related Go accuracy change when overriding well-learned Go responses (interaction *p* = .033). **(D)** Dual feedback improved goal-directed Go responses to novel associations (interaction *p* = .006). Error bars denote SEM. Color of bars reflects NoGo and Go stimulus colors.

Familiar condition: We tested the role of dual feedback in disrupting habitual control to familiar stimuli by performing a mixed-design repeated measures ANOVA on data from the Familiar condition, using NoGo accuracy as the DV. We found no main effect of Feedback, *F*(1,48) = 1.92, *p* = .173, η_p_^2^ = .04, a main effect of Color–Response_Mapping, *F*(1,48) = 28.12, *p* < .001, η_p_^2^ = .37, and a significant Feedback x Color–Response_Mapping interaction, *F*(1,48) = 8.48, *p* = .005, η_p_^2^ = .15 (see Fig 4A). This interaction suggests differential change in performance based on the availability of dual feedback, such that the presence of feedback resulted in a significantly smaller change in NoGo accuracy across mappings when managing familiar stimuli. Post-hoc t-tests confirmed a significant change in the No-Feedback group when switching from the congruent to the incongruent mapping, *t*(24) = 5.25, *p* < .001, replicating our findings from Experiments 1 and 2, but no significant change in the Feedback group *t*(24) = 1.92, *p* = .067. These results further corroborate the hypothesized effects of dual feedback on increasing outcome-sensitivity when managing Familiar associations.

Novel condition: To understand whether dual feedback enhanced goal-directed performance to newly-learned associations, we performed similar analyses on the Novel condition data. The mixed-design ANOVA, when controlling for Gender (which differed significantly across the Feedback groups in the Novel Stim_Familiarity condition, *χ2* (1) = 4.37, *p* = .037), yielded no main effect of Feedback, *F*(1,47) = 0.16, *p* = .899, η_p_^2^ < .01, or Color–Response_Mapping, *F*(1,47) = 0.78, *p* = .380, η_p_^2^ = .02; however, we found a near-significant Feedback x Color–Response_Mapping interaction on NoGo accuracy in the Novel condition: *F*(1,47) = 3.54, *p* = .066, η_p_^2^ = .07 (Fig 4B).

Our omnibus hierarchical regression model revealed Stim_Familiarity and Feedback regressors to be significant predictors of outcome-sensitivity. Combined, Stim_Familiarity and Feedback explained 26.6% of the variance in mapping-related NoGo accuracy change (β_Stim_Familiarity_ = -0.43, *p* < .001, β_Feedback_ = 0.28, *p* = .003; ΔR^2^ = .27). These data suggest that the differential mapping-related NoGo change observed in Experiment 2 was replicated in Experiment 3, and importantly, that dual feedback was able to significantly predict improvements in performance. The entirety of the omnibus test can be found in the supplement (S19 Table in S1 File). Taken together, we find strong evidence for beneficial effects of dual feedback on the balance between goal-directed and habitual control primarily when managing Familiar associations, and weaker evidence for when managing Novel associations.

#### Secondary index of outcome-sensitivity: Go accuracy

Familiar condition: As a supplementary assay of behavioral control via attentional processing, we analyzed Go accuracy using similar statistical procedures. We input Go accuracy as a DV, Feedback as a between-, and Color–Response_Mapping as a within-subjects factors into a mixed-design ANOVA. For the Familiar condition, we found a main effect of Feedback *F*(1,47) = 4.84, *p* = .033, η_p_^2^ = .09, a main effect of Color–Response_Mapping, *F*(1,47) = 4.94, *p* = .031, η_p_^2^ = .09, and a significant Feedback x Color–Response_Mapping interaction: *F*(1,47) = 4.81, *p* = .033, η_p_^2^ = .09 (Fig 4C), suggesting that Go accuracy was affected by dual feedback in the Familiar condition, in that it decreases significantly without the delivery of cumulative performance feedback paired with a monetary incentive. Post-hoc t-tests showed that Go accuracy was significantly lower in the incongruent mapping compared to the congruent mapping in the No-Feedback group, *t*(24) = 2.58, *p* = .017, yet no such difference was observed in the Feedback group, *t*(24) = 1.00, *p* = .925. These patterns lend support to the notion that dual feedback reallocates attention to disrupt habitual Go actions—our secondary assay of outcome-sensitivity.

Novel condition: We then tested the effect of dual feedback on Go accuracy in the Novel condition to determine whether our enhanced feedback manipulation improved goal-directed attentional processing when managing the contingency changes in newly-learned associations. We performed a mixed-design repeated measures ANOVA using Go accuracy as the DV, Feedback as the between-, and Color–Response_Mapping as the within-subjects factor, with Gender as a covariate (since Gender was significantly different across the two Feedback groups in the Novel Stim_Familiarity condition). We found no main effect of Feedback, *F*(1,47) = 3.50, *p* = .067, η_p_^2^ = .07, a main effect of Color–Response_Mapping, *F*(1,47) = 6.02, *p* = .018, η_p_^2^ = .11; and a significant Feedback x Color–Response_Mapping interaction: *F*(1,47) = 8.19, *p* = .006, η_p_^2^ = .15 (see Fig 4D). Post-hoc t-tests of each Feedback group confirms that monetary incentives paired with cumulative performance feedback significantly improved newly-learned Go associations that are executed by the goal-directed system: *t*(24) = -4.86, *p* < .001 with dual feedback, *t*(24) = -0.51, *p* = .616 with no feedback.

Our omnibus hierarchical regression model revealed that Stim_Familiarity and Feedback regressors significantly predict mapping-related Go accuracy changes. These regressors in sum accounted for 21% of the variance in the DV (β_Stim_Familiarity_ = -.36, *p* < .001, β_Feedback_ = .28, *p* = .004; ΔR^2^ = .21). These values suggest that Go accuracy is selectively impaired in the Familiar condition, and Feedback is able to promote goal-directed Go actions. Details of the omnibus regression can be found in the supplement (S20 Table in S1 File). Lastly, similar analyses performed with Go RT as DV yielded no significant findings (all *p*s > .05).

Finally, when we combine No-Feedback groups in Experiments 2 and 3 where participants undergo identical procedures, we find that the green–NoGo mapping (*M*_Green_ = 64.30, *SD*_Green_ = 20.35) yields significantly lower accuracy rates than the purple–NoGo mapping (*M*_Purple_ = 74.10, *SD*_Purple_ = 16.03), green vs. purple: *t*(98) = 2.67, *p* = .009. We compare green and purple directly, as these are the colors that the participants manage in the second half of the experiment in Experiments 2 and 3. With an increased sample size, we are able to detect that the incongruent color–response mapping yields impaired performance in comparison to the newly-learned color–response contingencies.

### Discussion

Collectively, our Experiment 3 findings suggest that a global motivational boost involving amplified performance and monetary feedback produces a habit-breaking effect that restores goal-directed control. Without feedback, we observe a significant change in NoGo and Go accuracy when familiar green and red light stimuli demand responses incongruent with daily experiences. We find that this outcome-insensitive habit (i.e., inflexible, cue-driven behavior that persists despite the outcome) of the green–go and red–stop actions is disrupted when participants are provided dual feedback, such that the significant incongruency-related NoGo change otherwise seen without feedback is prevented. Moreover, our dual feedback manipulation also improves goal-directed control when managing newly-learned associations, as evidenced by enhancements to NoGo and Go performance in the Novel group.

Although our task allows the study of well-established habits and their disruption, the mechanisms underlying the habit breaking process warrants further inquiry. Possibly, cumulative performance feedback may be enhancing intrinsic motivation to engage goal-directed control. The percentage score may provide individuals the opportunity to track task performance improvements, potentially boosting motivation to improve task-competence [41]. The delivery of a monetary incentive may be engaging extrinsic motivation, making the goal of accurate performance more salient, thus shifting attentional and motivational processes to the consequences of behavior to recruit goal-directed control. It is also possible that although not explicitly instructed, participants may have anticipated a future cash bonus, leading to a stronger link between actions and their consequences. Such instructions were provided in another study, yielding comparable findings [42]. It is important to note, however, that all participants, in all stages of the task, received trial-by-trial feedback (i.e., correct or incorrect). Therefore, the performance change effects observed following cumulative performance and monetary feedback delivery cannot be attributed to trial-by-trial feedback. Finally, the cash bonus was provided by the experimenter, possibly engaging social approval motives. Given the multiple possible factors, our interpretations of why the dual feedback disrupts habitual associations is speculative, and further research is warranted to elucidate the underpinnings of habit breaking. In short, the dual feedback provided in our experiment may be producing a global increase in motivation, resulting in more deliberate control of otherwise inflexible behaviors.

Importantly, the beneficial effect of such feedback generalizes to more flexible goal-directed performance, as we observe a significant improvement in Go accuracy (and slight improvement in NoGo accuracy) scores to novel blue–go and purple–stop contingencies when participants are provided dual feedback. Without feedback, we find no mapping-related difference in accuracy to novel stimuli, serving as support for the flexible nature of these newly-learned associations that can readily be reassigned per changes in one’s environment. These findings identify dual feedback as a powerful predictor of motivational control enhancement.

Lastly, we conducted a pooled analysis using identically obtained data from Experiments 2 and 3 to follow up on the expected effect of diminished green–NoGo accuracy vs. novel associations. We note that we cannot attribute the absence of the expected facilitation of Go responding to green versus other colors effect in Experiment 1 to insufficient power with certainty. Although we determined a sample size based on a power estimation of 80%, it is also important to acknowledge the possibility of type II errors. We may have been sufficiently powered to detect the effect of red signal providing an advantage over other colors in NoGo accuracy, but perhaps underpowered to find the effect for green–NoGo eliciting poorer performance compared to other colors. The pooled analyses here, and another study with a within-subject design with more participants [42] suggest that the significant green–NoGo performance decrement compared to other associations may be detected with a larger sample size.

## General discussion

In a three-experiment study, we introduce a novel Go/NoGo task that capitalizes on familiar, well stamped-in associations of red–stop and green–go to elicit habitual control, and establish dual feedback (i.e., monetary reward paired with cumulative performance tracking) as an intervention to break these well-learned habits to restore goal-directed control. The familiar stimuli in our task evoke a color–response habit that is evident in our participants’ difficulty overriding the well-established associations. We found that the familiar stimuli yield persistent instrumental responses even when these contingencies are manipulated to render green–go and red–stop color–responses disadvantageous for task performance. We also report enhanced goal-directed control (i.e., a disruption of the color–response habits) due to dual feedback, lending support to the effectiveness and scope of our performance enhancing feedback manipulation.

Accordingly, an important goal of our study was to establish our paradigm as a tool that captures real-world habits. Similar to the traditional habit task in rodents [5], our task explicitly involves learning the contingencies among a stimulus (i.e., the Go/NoGo signals), a response (i.e., button press), and an outcome (i.e., trial-by-trial correct/incorrect slide). However, in contrast to the traditional habit task [1,5,11,18,32], we capitalized on stimuli with well-established real-world associations to determine whether responses congruent with daily experiences persist despite a change in stimulus-response contingencies. Thus, we did not need to develop stimulus-response associations in a lengthy training session while hungry, devalue an appetitive outcome (e.g. selective satiety to food), and then test responses in a brief, unrewarded extinction session while sated. Instead, we were able to compare behavioral performance across opposite color–response mapping phases that were as close as possible in experimental contexts. In Experiment 1, we demonstrated the rigidity of the familiar green–go and red–stop contingencies compared to the newly-learned, flexible associations. The outcome-insensitive responses elicited by the familiar stimuli were reflected by a significant mapping-related change not observed when participants managed novel stimuli. Specifically, participants had less difficulty with the red–NoGo association in relation to green–NoGo, whereas variations in color–response mappings did not produce significant differences when managing novel associations (e.g., blue–NoGo or purple–NoGo). It is worth mentioning that the habits demonstrated here are not effector specific [43], in that we do not assert whether red and green light stimuli trigger actions that are alike to those that may be triggered in a driving context (e.g., a foot-press response at red, or foot-release at green). Rather, the familiar stimuli used in our task may be evoking a general approach and avoid response, which, in the context of the task, is mapped onto Go and NoGo responses.

Habit disruption is a translationally valuable research avenue seldomly examined in the habit literature. Studies that demonstrated feedback-driven improvements in a wide range of cognitive functions motivated us to implement a feedback manipulation into our well-learned habit eliciting task to achieve habit disruption. Particularly relevant are studies that have used feedback in the form of monetary incentives to boost task-switching performance [26,30]. We speculate that the improvement of goal-directed control may be due to an amplification of outcome representations due to the enhanced link between responses and their consequences once the stakes have been raised. This explanation is in agreement with the notion that monetary rewards engage top-down control, effectively improving task performance in task-switching [30]. Furthermore, although we had not promised the prospect of a future reward during monetary bonus delivery, previous research has demonstrated that the mere prospect of future rewards may boost task-switching performance [28]. Although task-switching processes are not entirely analogous with the shift between habitual and goal-directed control, because they have been show to engage top-down control over actions via feedback [30], we believe these studies share mechanistic components, particularly in relation to cognitive control. Future studies of habit disruption can dissociate the specific neural processes involved in the reemergence of control, potentially via the cognitive control network that is associated with goal-directed behaviors [44].

Certainly, a Go/NoGo task like ours also captures mental processes other than the control of motivated behaviors, such as response inhibition. However, here we introduce stimuli and Go/NoGo contingencies that are further separated by familiarity. Importantly, other studies have not manipulated cue-response-outcome associations to test their flexibility. Many have used these colors as Go or NoGo signals that are intuitive to the participant (which we believe further speaks to the notion that these stimuli indeed hold real-world meaning) [19–21]. A study has even investigated whether green and red signals facilitate go and stop behaviors in a stop-signal task and found supporting evidence for the effect of environmental familiarity and inhibitory control [22]. However, ours is the first study that examines these familiarities in the perspective of habits and goals. We probe these well-learned associations further than their past use for convenience and ask: 1) do these signals elicit inflexible, habitual behaviors that prevail regardless of task instructions compared to newly-learned associations, and 2) can we protect individuals from the inflexible responding evoked by these signals when one must override the well-learned meanings associated with these stimuli? Accordingly, if changing color–response mappings *only* captured response inhibition, this would not explain the flexible performance to novel stimuli.

We tested the strength of the habits evoked in our paradigm by introducing a motivation-based intervention: cumulative performance feedback. This type of feedback was not successful in preventing habitual control, supporting the notion that these existing habits are rigid enough to prevail even in the face of a motivational intervention. Nonetheless, performance feedback was able to produce promising results via secondary assays of behavioral flexibility. Namely, the prevention of habitual “Go” actions, possibly by performance feedback adaptively reallocating attention to Go stimuli, motivated the augmentation of our feedback manipulation to amplify its effect on behavioral flexibility. In Experiment 3, our combined delivery of performance and monetary feedback prevented the mapping-related change that is the result of a habit-dominated action control system, possibly improving goal-directed control by enhancing the salience of the outcome. In sum, we demonstrated well-existing habits, tested the limits of their associative strength, and provided the foundation for better understanding the restoration of goal-directed control.

Many habit paradigms that emulate the outcome-insensitive nature of habits have in common a shortcoming that limits generalizability to the typical habit experience: difficulty capturing well-learned habits in the lab that can provide a platform for studying habit disruption. Habit strength is limited by the participants’ brief exposure to experimental paradigms, and targeting these behaviors that are rendered inflexible in the lab may not be representative of habits encountered in the real world [18]. Perhaps due to these difficulties, well-learned habits and habit disruption research have been relatively better-represented in field experiments compared to the laboratory setting. For example, several field studies have examined the efficacy of interventions to change various presentations of daily habits, such as recycling and snacking habits [45–47]. However, recent efforts to bridge lab and field experiments have shown promising results. Although not an experiment of habit disruption, in a recent report, the slips-of-action task in the lab was examined alongside a more ecologically-relevant representation of habits—namely the habit of using one’s house keys. In this study, participants demonstrated an outcome-insensitive habit by making key choice errors, such that they persisted in choosing the incorrect key following a change in key covers. The attentional underpinnings of this behavior significantly correlated with slips of action performance, underlining the importance of focusing on well-established behaviors for an improved empirical approach to habit research [48].

One strategy that has proven beneficial in tackling habit change is implementation intentions, which provides individuals with an if-then plan (i.e., “if X happens, I will do Y”; or in a lab task, “if I see stimulus X, I will press Y”)—an aid to override unwanted or inflexible behaviors [49]. In the lab, implementation intentions have produced promising results, albeit with limited efficacy in disrupting strong habits. For instance, Webb and colleagues trained participants for five days on a target detection task, and successfully disrupted this lab-automated association using implementation intentions. However, this planning strategy did not break unwanted smoking habits, lending credence to the idea that the experimental resources at our disposal may not be sufficient in effectively stopping well-established habits [50]. Although this study approached habitual control from an attentional rather than a value-driven perspective, paralleling evidence from the motivational control literature has recently been reported. In another lab study, Verhoeven et al. employed planning strategies within a single experimental session to reduce action slips in an outcome-devaluation task [51]. Implementation intentions were more effective than goal-intentions (an outcome-based planning strategy, such as “I will not press for outcome X”) in reducing action slips when managing abstract images as outcomes, suggesting that implementation intentions may serve as a promising strategy in studying habit disruption—however, effective paradigms to demonstrate well-learned, outcome-insensitive habits, and an intervention to disrupt them are needed. In our study, we developed a task that allowed us to directly capture ecologically significant, well-established habits via the familiar green–go and red–stop associations. We present our Go/NoGo task with familiar and novel stimuli as a strong candidate for demonstrating habitual behaviors—bridging the success of field studies and the rigor and controllability of lab experimentation. We also illustrate that a salient feedback-based intervention may be utilized to shift cue-driven performance to become value-driven, laying the foundation to translational applications.

Our work also asserts that the use of familiar stimuli may circumvent the obstacles of training length and stimulus–response strength in habit research—an important step in improving paradigms to foster effective habit disruption strategies. A few prior studies have considered a similar approach. In a study investigating habits in substance use disorder, McKim and colleagues induced stimulus familiarity by pre-training a set of stimuli, and tested the strength of the familiar versus novel stimulus sets on a subsequent day via the reversal of a subset of these contingencies [17]. They found that compared to healthy controls, individuals with substance use disorder performed better in well-learned stimulus–response execution, yet exhibited impairments in managing contingency reversal. In accord with these findings, our study reveals that when managing contingencies that have been well-established throughout development—beyond an experimental pre-training stage—the recruitment of the habit system may also be evident in healthy individuals. Similarly, developmental and clinical researchers have used familiar green and red stimuli in Go/NoGo tasks with children suffering from attention-deficit/hyperactivity disorder, as well as healthy adults to reduce task demands, and justified their decision by identifying these colors as having developmental relevance [19,20]. These prior reports highlight the utility of capitalizing on existing associations when examining habits, especially for clinical examinations of behavioral rigidity. Thus, we further contribute to the literature by introducing a task that requires minimal familiarity training, and by the inclusion of a motivational strategy to disrupt the familiarity-driven outcome-insensitivity. These contributions may be especially useful for optimizing costly fMRI designs, and benefit future translational neuroscience work that aims to reveal the neural bases of habit disruption.

Certain limitations of our study should be considered in future examinations of habit expression and disruption. Although we counterbalanced the order of mapping presentation (e.g., whether participants managed the red–stop/green–go mapping first) in Experiment 1 to demonstrate well-learned habit expression, we did not counterbalance the mapping phases in Experiments 2 and 3 when attempting habit disruption. We reasoned that presenting the congruent mapping first, then providing the feedback manipulation, would allow us to detect whether feedback prevented accuracy changes that were observed in the No-Feedback groups (as well as in Experiment 1). However, testing the extent of feedback efficacy could be probed in future research by counterbalancing the order of color–response mappings that are separated by feedback. Possibly, individuals who manage first the color–response mappings that are incongruent with daily experiences (e.g., green-stop) would experience a greater boost in accuracy when switching to the congruent mapping (e.g., red–stop) after receiving dual feedback compared to individuals who received no feedback. This counterbalancing would also permit a more direct replication of Experiment 1’s findings (i.e., illustrating again the finding that the habit expression effect is not affected by order of mapping presentation).

Additionally, if these familiar red and green stimuli elicit outcome-insensitive habits, it may be argued that color–response mappings that are incongruent with daily experiences should display the lowest accuracy rates. However, in Experiment 1, green–NoGo accuracy was comparable to that of blue–NoGo or purple–NoGo mappings. A similar pattern has been documented in previous research: Hochman et al. found that better stop-signal task performance using red and green light stimuli was primarily driven by a red–stop association as opposed to a green–go association [22]. In light of this report, it could be the case that a green–NoGo impairment is more difficult to detect than the advantage a red–stop association may elicit. Although we attempted to power our study adequately, our power analysis of Experiment 1 was based on pilot data from 6 individuals. Additionally, we conducted the power analysis using the effect size of the primary Stim_Familiarity x Color–Response_Mapping interaction of interest. Although this was an appropriate estimate for detecting the habit expression effect elicited by the well-learned stimuli (i.e., the difference between green and red–NoGo compared to the difference between blue and purple–NoGo), we may not have been powered enough to detect specific differences between green–NoGo and purple–NoGo associations. To test whether a larger sample size would allow us to detect a significant green–NoGo impairment effect, we combined the data from the No-Feedback groups in Experiments 2 and 3 (n = 100 No-Feedback participants), where participants underwent comparable procedures. Given this larger sample size, we found that the green–NoGo mapping indeed produced significantly lower accuracy rates than the corresponding novel color–response mapping (purple–NoGo). Furthermore, in a version of this task that employs a within-subject design in which all participants manage familiar and novel Go/NoGo contingencies, we indeed report significantly lower accuracy rates to green as a NoGo stimulus compared to novel associations [42].

## Conclusions

The disproportionate focus on habit formation and expression in the literature motivated us to direct our efforts to an area of habit research that has been less-explored: habit disruption. Although much research now confirms the habitual aspects of various pathologies, studies examining the restoration of these behavioral rigidities are relatively scarce. Here, we introduce a task that allows us to examine a more complete signature of motivational control by capturing well-learned habits and newly-learned goal-directed behaviors, as well as the possibility to test manipulations that may restore deliberate control. This method may be especially beneficial for understanding the neural markers of habits in healthy and compromised populations, as it capitalizes on existing associations that do not require extended lab-training. We also underline the efficacy of feedback in disrupting well-learned habits and promoting outcome-driven, goal-directed behaviors. This motivation-based manipulation may further inform the mechanisms underlying the habit disruption process—a translationally valuable research domain with direct clinical relevance.

## Supporting information

S1 File (DOCX)

S1 Data (ZIP)
