# Supplementary material for: Demonstrating and disrupting well-learned habits
Source: PLoS One. 2020 Jun 12;15(6):e0234424. doi: 10.1371/journal.pone.0234424 (PMC7292414; doi:10.1371/journal.pone.0234424)
Supplement: S1 File — (DOCX) [file pone.0234424.s001.docx]

**Supplement**

**Experiment 1**

**Bayesian analysis of outcome-sensitivity: NoGo accuracy**

We derived Bayes factors (BF_10_) for Stim_Familiarity and Color–Response_Mapping main effects and their interaction to determine how much evidence our data provided in support of the Stim_Familiarity x Color–Response_Mapping interaction model for each DV over the null hypothesis and models that solely included the main effects. A BF_10_ of 1 indicates equal support for the alternate and null hypotheses. BF_10_ < 1 provides more support for the null, and BF_10_ > 1 provides more support for the alternate hypothesis. We interpreted BF_10_ > 3 as supporting moderate evidence for the alternate hypothesis, and interpreted BF_10_ < 0.33 as moderate evidence for the null hypothesis. Similarly, we interpreted BF_10_ > 10 to be strong evidence for the alternate hypothesis, and BF_10_ < 0.1 to be strong evidence for the null hypothesis [1,2]. We then divided the interaction’s BF_10_ by the best performing main effects model’s BF_10_ to quantify how much more likely the data fit our hypothesis of an interaction model that suggests differential mapping-related accuracy change across the Familiar and Novel Stim_Familiarity conditions over the next best model.

A Jeffreys-Zellner-Siow Bayes factor ANOVA [3] of NoGo accuracy that closely follows our primary mixed-design ANOVA in the main text suggests that the data provided moderate evidence in support of the hypothesized Stim_Familiarity x Color–Response_Mapping interaction model (BF_10_ = 4.12) over the best performing main effects model (BF_10_ = 0.39). These results indicate that the data are 4.12 times more likely to fit the hypothesized interaction model that describes a differential mapping-related accuracy change across the Familiar and Novel Stim_Familiarity conditions over the null hypothesis. The data are also 10.56 times more likely to fit the next best-fitting main effects model (see S1 Table).

**S1 Table.** **Bayesian analyses of outcome-sensitivity across Familiar and Novel Stim_Familiarity conditions: NoGo accuracy.**

| **Model Comparison** | | | | | | | | | | | |
| --- | --- | --- | --- | --- | --- | --- | --- | --- | --- | --- | --- |
| **Models** | | **P(M)** | | **P(M\|data)** | | **BF _M_** | | **BF _10_** | | **error %** | |
| Null model (incl. subject) |  | 0.200 |  | 0.148 |  | 0.697 |  | 1.000 |  |  |  |
| Map. + Stim_Fam. + Map. x Stim_Fam. |  | 0.200 |  | 0.612 |  | 6.305 |  | 4.123 |  | 2.567 |  |
| Map. |  | 0.200 |  | 0.094 |  | 0.416 |  | 0.635 |  | 1.025 |  |
| Stim_Fam. |  | 0.200 |  | 0.088 |  | 0.387 |  | 0.594 |  | 1.708 |  |
| Map. + Stim_Fam. |  | 0.200 |  | 0.057 |  | 0.244 |  | 0.387 |  | 1.899 |  |
|  | | | | | | | | | | | |
| *Note.**All models include subject. Non-null models listed in order of BF_10_ . Map.: Color–Response_Mapping, Stim_Fam: Stim_Familiarity. BF_M_: Bayes factor of the change from prior to posterior model odds.* | | | | | | | | | | | |

**Omnibus regression to confirm mapping-related accuracy change – Primary measure of outcome-sensitivity: NoGo accuracy**

The omnibus regression closely follows the primary mixed-design ANOVAs outlined in the main text (Experiment 1 Methods), and simply reduces these multiple ANOVAs to a single regression per DV (e.g., a regression with NoGo accuracy difference across Color–Response Mappings as DV, Stim_Familiarity and controlled variables as fixed factors). These confirmatory omnibus regressions provide additional information on the controlled Age, Gender, and Impulsivity variables (regardless of whether group differences are evident across groups) while strengthening the validity of the primary ANOVAs.

We derived a ΔNoGo_Accuracy (i.e., change in NoGo accuracy scores across Color–Response_Mappings) DV to quantify the mapping-related change for each subject. This ΔNoGo_Accuracy variable serves as the primary measure of outcome-sensitivity, in that a greater change represents greater outcome-insensitivity. Specifically, difficulty overriding the Familiar red–NoGo association for the green–NoGo association indicates a cue-driven habit. In contrast, we would not expect pronounced ΔNoGo_Accuracy change when participants manage Novel NoGo contingencies (i.e., blue–NoGo and purple–NoGo should yield similar accuracy scores). Participants with DV standardized residual values below -3.3 and above +3.3 were identified as outliers [4]. In such cases, we performed identical analyses without outlier participants to verify robustness of findings, but only report these excluded analyses if outliers produced substantial changes in statistical significance.

We employed a hierarchical multiple regression model to extract the predictive strength of the between-group Stim_Familiarity variable while controlling for Age, Gender, Order of Color–Response_Mapping phase (i.e., whether a subject completed a particular color–response mapping first), and Impulsivity. We entered the controlled Age, Gender, Order, and Impulsivity regressors into the first, and the Stim_Familiarity regressor of interest into the second step of the model. Therefore, our hierarchical multiple regression model yielded an R^2^ change value (ΔR^2^) for Stim_Familiarity, determining whether mapping-related changes are predicted specifically by Stim_Familiarity (i.e., contingency change in Familiar versus Novel stimuli). We also derived the corresponding *F*_change_ value, which compares the predictive strength of the variables in the second step of the model with those in the first step (i.e., confirming whether ΔR^2^ reflects a significant change in the model’s predictive strength).

The regression model met the assumptions of normality and homoscedasticity. Multicollinearity tests produced negligible Variance Inflation Factors (VIF), confirming linearity assumptions of the regression (VIF for all variables < 1.09). Model 1, a linear combination of the controlled variables of Age, Gender, Order, and Impulsivity, did not significantly predict outcome-sensitivity: *F*(4,45) = 0.46, *p* = .767, and only explained 4% of the variance in ΔNoGo_Accuracy (R^2^ = .04). Additionally, no controlled regressor independently predicted a change in ΔNoGo_Accuracy (all β coefficient *p’*s. > .05). In the second step of the regression, the inclusion of Stim_Familiarity as a regressor explained an additional 15.5% of the variance in outcome-sensitivity: β_Stim_Familiarity_ = -.40, ΔR^2^ = .15, *F*_change_ (1,44) = 8.47, *p* = .006—a significant contribution. Thus, the addition of the significant Stim_Familiarity regressor rendered the entirety of Model 2 a near-significant predictor of outcome-sensitivity, despite the null contributions from Age, Gender, Order, and Impulsivity: *F*(5,44) = 2.12, *p* = .081 (see S2 Table).

**S2 Table. Summary of the Hierarchical Multiple Regression Model for Outcome-Insensitivity as Assayed by ΔNoGo_Accuracy.**

| Variable | *Toler.* | *VIF* | *B* | *SE* | *β* | *t* | *sig.* |
| --- | --- | --- | --- | --- | --- | --- | --- |
| **Model 1** |  |  |  |  |  |  |  |
| Age | .98 | 1.02 | 0.57 | 0.82 | .10 | 0.69 | .493 |
| Gender | .97 | 1.04 | -2.69 | 5.08 | -.08 | -0.53 | .599 |
| Impulsivity | .93 | 1.07 | -0.25 | 0.35 | -.11 | -0.71 | .483 |
| Order | .96 | 1.04 | 3.12 | 4.88 | .95 | 0.64 | .527 |
| **Model 2** |  |  |  |  |  |  |  |
| Age | .97 | 1.03 | 0.34 | 0.77 | .06 | 0.44 | .662 |
| Gender | .96 | 1.05 | -1.30 | 4.73 | -.04 | -0.27 | .784 |
| Impulsivity | .93 | 1.08 | -0.33 | 0.33 | -.14 | -0.99 | .326 |
| Order | .96 | 1.04 | 2.88 | 4.52 | .09 | 0.64 | .527 |
| Stim_Familiarity | .98 | 1.02 | -13.06 | 4.50 | -.40^**^ | -2.91 | **.006** |

| Model Summary Statistics | | | | | | |
| --- | --- | --- | --- | --- | --- | --- |
| Model | *R^2^* | *F* | *F sig.* | *ΔR^2^* | *F*_change_ | *F*_change_ *sig.* |
| **Model 1** | .04 | 0.46 | .767 |  |  |  |
| **Model 2** | .19 | 2.12 | .081 | .15 | 8.47 | **.006** |

*Note:* *Top layer of table depicts all regressors included in the hierarchical model and their respective statistics. Bottom layer of table, Model Summary Statistics, depicts the predictive strength of each model. Delta R^2^ (ΔR^2^) and corresponding F_change_ values denote the specific improvement of Model 2 over Model 1 in predicting the dependent variable. Toler. = Tolerance; VIF = Variance Inflation Factor. Significant p-values (alpha = .05) depicted in bold typeface.*

These results also suggest that the differential mapping-related change observed across Familiar and Novel Stim_Familiarity conditions is not due to the order in which participants managed color–response mappings. The Order variable did not significantly predict ΔNoGo_Accuracy in our model (β = .09, *p* = .527). We found no interaction between factors of Order and Color–Response_Mapping in NoGo accuracy as a result of the repeated measures ANOVA: *F*(1,48) = 0.35, *p* = .555, η_p_^2^ < .01. We performed the same ANOVA separately in Familiar and Novel Stim_Familiarity conditions and observed no significant interactions in either group (*p*s > .05).

**Bayesian analysis of outcome-sensitivity: Go accuracy**

A Bayes factor ANOVA of Go accuracy that closely follows the reported mixed-design ANOVA suggests that the data did not provide substantial evidence in support of the hypothesized Stim_Familiarity x Color–Response_Mapping interaction model (BF_10_ = 0.38) over the best performing main effects model (BF_10_ = 0.54). These results indicate that the data are 1.42 times more likely to fit the main effects model than our hypothesized interaction model of differential mapping-related Go accuracy change across the Familiar and Novel Stim_Familiarity conditions (see S3 Table).

**S3 Table. Bayesian analyses of outcome-sensitivity across Familiar and Novel Stim_Familiarity conditions: Go accuracy.**

| **Model Comparison** | | | | | | | | | | | |
| --- | --- | --- | --- | --- | --- | --- | --- | --- | --- | --- | --- |
| **Models** | | **P(M)** | | **P(M\|data)** | | **BF _M_** | | **BF _10_** | | **error %** | |
| Null model (incl. subject) |  | 0.200 |  | 0.287 |  | 1.609 |  | 1.000 |  |  |  |
| Map. |  | 0.200 |  | 0.303 |  | 1.737 |  | 1.055 |  | 1.765 |  |
| Map. + Stim_Fam. |  | 0.200 |  | 0.155 |  | 0.735 |  | 0.541 |  | 3.107 |  |
| Stim_Fam. |  | 0.200 |  | 0.147 |  | 0.687 |  | 0.511 |  | 1.262 |  |
| Map. + Stim_Fam. + Map. x Stim_Fam. |  | 0.200 |  | 0.108 |  | 0.487 |  | 0.378 |  | 2.871 |  |
|  | | | | | | | | | | | |
| *Note.**All models include subject. Non-null models listed in order of BF_10_ . Map.: Color–Response_Mapping, Stim_Fam: Stim_Familiarity.* *BF_M_: Bayes factor of the change from prior to posterior model odds.* | | | | | | | | | | | |

**Omnibus regression to confirm mapping-related accuracy change – Secondary measure of outcome-sensitivity: Go accuracy**

We performed an identical omnibus regression using ΔGo_Accuracy as DV—the secondary assay of outcome-sensitivity. The regression model met the assumptions of normality and homoscedasticity. Multicollinearity tests produced negligible Variance Inflation Factors (VIF), confirming the assumption of linearity in the regression model (VIF for all variables < 1.09).

Collectively, the linear combination of Age, Gender, Order, and Impulsivity did not significantly predict mapping-related changes in Go accuracy: *F*(4,45) = 2.18, *p* = .087. As depicted in S4 Table, closer examination of the individual regressors revealed a significant role played by Age, such that older participants suffered a greater mapping-related Go accuracy change: β_Age_ = -.31, *p* = .027. The inclusion of the Stim_Familiarity regressor significantly improved the predictive strength of the model in step 2: β_Stim_Familiarity_ = -.27, ΔR^2^ = .07, *F*_change_(1,44) = 4.10, *p* = .049, and the significant contribution of Age remained: β_Age_ = -.34, *p* = .014. Although age was a significant predictor of change in Go accuracy, because we had no *a priori* hypothesis, and the correlational direction of this relationship varied across Stim_Familiarity conditions (Familiar Stim_Familiarity Pearson’s *r* = .43; Novel Stim_Familiarity Pearson’s *r* = -.70), we refrain from further age-related speculation.

**S4 Table. Summary of the Hierarchical Multiple Regression Model for Outcome-Insensitivity as Assayed by ΔGo_Accuracy.**

| Variable | *Toler.* | *VIF* | *B* | *SE* | *β* | *t* | *sig.* |
| --- | --- | --- | --- | --- | --- | --- | --- |
| **Model 1** |  |  |  |  |  |  |  |
| Age | .98 | 1.02 | -0.60 | 0.26 | -.31 | -2.29^*^ | **.027** |
| Gender | .97 | 1.04 | 1.83 | 1.61 | .16 | 1.14 | .261 |
| Impulsivity | .93 | 1.07 | -0.02 | 0.11 | -.03 | -0.20 | .843 |
| Order | .96 | 1.04 | -2.02 | 1.55 | -.18 | -1.30 | .199 |
| **Model 2** |  |  |  |  |  |  |  |
| Age | .97 | 1.03 | -0.65 | 0.25 | -.34^*^ | -2.56 | **.014** |
| Gender | .96 | 1.05 | 2.15 | 1.56 | .19 | 1.38 | .176 |
| Impulsivity | .93 | 1.08 | -0.04 | 0.11 | -.05 | -0.37 | .716 |
| Order | .96 | 1.04 | -2.07 | 1.50 | -.19 | -1.38 | .174 |
| Stim_Familiarity | .98 | 1.02 | -3.01 | 1.49 | -.27^**^ | -2.03 | **.049** |

| Model Summary Statistics | | | | | | |
| --- | --- | --- | --- | --- | --- | --- |
| Model | *R^2^* | *F* | *F sig.* | *ΔR^2^* | *F*_change_ | *F*_change_ *sig.* |
| **Model 1** | .16 | 2.18 | .087 |  |  |  |
| **Model 2** | .23 | 2.68 | **.034** | .07 | 4.10 | **.049** |

*Note: Top layer of table depicts all regressors included in the hierarchical model and their respective statistics. Bottom layer of table, Model Summary Statistics, depicts the predictive strength of each model. Delta R^2^ (ΔR^2^) and corresponding F_change_ values denote the specific improvement of Model 2 over Model 1 in predicting the dependent variable. Toler. = Tolerance; VIF = Variance Inflation Factor. Significant p-values (alpha = .05) depicted in bold typeface.*

Similar to our primary assay of outcome-sensitivity, change in Go accuracy was not due to the order in which participants managed color–response mappings (β_Order_ = -.19, *p* = .174). There was no significant Order x Color–Response_Mapping interaction in Go accuracy: *F*(1,48) = 2.26, *p* = .140, η_p_^2^ = .04. We performed the same ANOVA separately in Familiar and Novel Stim_Familiarity conditions and observed no significant interactions in either group (*p*’s > .05).

**Experiment 2**

**Replication of Experiment 1 via Bayesian analyses of outcome-sensitivity**

We derived BF_10_ for Stim_Familiarity and Color–Response_Mapping main effects and their interaction for each DV to replicate our findings from Experiment 1. We also performed similar Bayes factor ANOVAs to capture the role of cumulative performance feedback on mapping-related accuracy change. This approach permitted us to observe the evidence our data provided in support of the hypothesized Feedback x Color–Response_Mapping interaction model (controlling for any covariate that differed across groups) over models that solely included the main effects. We then divided the interaction’s BF_10_ by the main effects’ BF_10_ to quantify the likelihood of the interaction model in line with our cumulative performance feedback hypothesis fitting the data over the main effects’ models.

The Bayes factor ANOVA of NoGo accuracy that closely follows Experiment 1’s primary mixed-design ANOVA suggests that the data provided moderate evidence in support of the hypothesized Stim_Familiarity x Color–Response_Mapping interaction model (BF_10_ = 3.17) over the main effects model (BF_10_ = 2.59). These results indicate that the data are 3.17 times more likely under the hypothesized interaction model compared to the null hypothesis, and this interaction model is also 1.22 times more likely to fit the data compared to the best fitting main effects model (see S5 Table).

**S5 Table. Replication of Experiment 1 effect via Bayesian analyses of outcome-sensitivity: NoGo accuracy (No-Feedback group only).**

| **Model Comparison** | | | | | | | | | | | |
| --- | --- | --- | --- | --- | --- | --- | --- | --- | --- | --- | --- |
| **Models** | | **P(M)** | | **P(M\|data)** | | **BF _M_** | | **BF _10_** | | **error %** | |
| Null model (incl. subject) |  | 0.200 |  | 0.068 |  | 0.291 |  | 1.000 |  |  |  |
| Map. |  | 0.200 |  | 0.520 |  | 4.329 |  | 7.673 |  | 1.454 |  |
| Map. + Stim_Fam. + Map. x Stim_Fam. |  | 0.200 |  | 0.215 |  | 1.095 |  | 3.173 |  | 1.407 |  |
| Map. + Stim_Fam. |  | 0.200 |  | 0.175 |  | 0.850 |  | 2.588 |  | 2.032 |  |
| Stim_Fam. |  | 0.200 |  | 0.022 |  | 0.091 |  | 0.329 |  | 3.294 |  |
|  | | | | | | | | | | | |
| *Note.  All models include subject. Non-null models listed in order of BF_10_ . Map.: Color–Response_Mapping, Stim_Fam: Stim_Familiarity. BF_M_: Bayes factor of the change from prior to posterior model odds.* | | | | | | | | | | | |

A Bayes factor ANOVA of Go accuracy indicated that the data provided moderate evidence in support of the hypothesized Stim_Familiarity x Color–Response_Mapping interaction model (BF_10_ = 6.94) over the main effects model (BF_10_ = 4.97). These results indicate that the data are 6.94 times more likely under the hypothesized interaction model compared to the null hypothesis, and this interaction model is also 1.40 times more likely to fit the data compared to the best fitting main effects model (see S6 Table).

**S6 Table. Replication of Experiment 1 effect via Bayesian analyses of outcome-sensitivity: Go accuracy (No-Feedback group only).**

| **Model Comparison** | | | | | | | | | | | |
| --- | --- | --- | --- | --- | --- | --- | --- | --- | --- | --- | --- |
| **Models** | | **P(M)** | | **P(M\|data)** | | **BF _M_** | | **BF _10_** | | **error %** | |
| Null model (incl. subject) |  | 0.200 |  | 0.066 |  | 0.282 |  | 1.000 |  |  |  |
| Map. + Stim_Fam. + Map. x Stim_Fam |  | 0.200 |  | 0.457 |  | 3.369 |  | 6.944 |  | 1.655 |  |
| Map. |  | 0.200 |  | 0.327 |  | 1.945 |  | 4.970 |  | 1.136 |  |
| Map. + Stim_Fam. |  | 0.200 |  | 0.125 |  | 0.571 |  | 1.897 |  | 1.369 |  |
| Stim_Fam. |  | 0.200 |  | 0.025 |  | 0.102 |  | 0.378 |  | 0.834 |  |
|  | | | | | | | | | | | |
| *Note.  All models include subject. Non-null models listed in order of BF_10_ . Map.: Color–Response_Mapping, Stim_Fam: Stim_Familiarity. BF_M_: Bayes factor of the change from prior to posterior model odds.* | | | | | | | | | | | |

**Bayesian analyses of the effects of performance feedback: NoGo accuracy**

We conducted a Bayes factor ANOVA of NoGo accuracy that closely follows the reported mixed-design ANOVA. Although the data were 26.55 times more likely under the hypothesized Feedback x Color–Response_Mapping interaction model (BF_10_ = 26.55) over the null hypothesis, the best performing main effects model of Color–Response_Mapping was substantially better fitting (BF_10_ = 180.03; 6.78 times more likely than the interaction model; see S7 Table).

**S7 Table.** **Bayesian analyses of the effects of performance feedback on Familiar Stim_Familiarity condition outcome-sensitivity: NoGo accuracy**

| **Model Comparison** | | | | | | | | | | | |
| --- | --- | --- | --- | --- | --- | --- | --- | --- | --- | --- | --- |
| **Models** | | **P(M)** | | **P(M\|data)** | | **BF _M_** | | **BF _10_** | | **error %** | |
| Null model (incl. subject) |  | 0.200 |  | 0.003 |  | 0.014 |  | 1.000 |  |  |  |
| Map. |  | 0.200 |  | 0.625 |  | 6.674 |  | 180.026 |  | 1.556 |  |
| Map. + Fb. |  | 0.200 |  | 0.278 |  | 1.537 |  | 79.930 |  | 1.051 |  |
| Map. + Fb. + Map. x  Fb. |  | 0.200 |  | 0.092 |  | 0.406 |  | 26.553 |  | 1.644 |  |
| Fb. |  | 0.200 |  | 0.001 |  | 0.006 |  | 0.411 |  | 0.608 |  |
|  | | | | | | | | | | | |
| *Note.  All models include subject. Non-null models listed in order of BF_10_ . Map.: Color–Response_Mapping, Fb.: Feedback. BF_M_: Bayes factor of the change from prior to posterior model odds.* | | | | | | | | | | | |

Furthermore, we conducted a Bayes factor ANOVA of NoGo accuracy in the Novel Stim_Familiarity condition that closely follows the reported mixed-design ANOVA. Our Age-corrected Feedback x Color–Response_Mapping intraction model yielded strong evidence for the null hypothesis (BF_10_ = 0.09; see S8 Table).

**S8 Table. Bayesian analyses of the effects of performance feedback on Novel Stim_Familiarity condition outcome-sensitivity: NoGo accuracy**

| **Model Comparison** | | | | | | | | | | | |
| --- | --- | --- | --- | --- | --- | --- | --- | --- | --- | --- | --- |
| **Models** | | **P(M)** | | **P(M\|data)** | | **BF _M_** | | **BF _10_** | | **error %** | |
| Null model (incl. subject) |  | 0.100 |  | 0.293 |  | 3.728 |  | 1.000 |  |  |  |
| Age |  | 0.100 |  | 0.262 |  | 3.203 |  | 0.896 |  | 1.951 |  |
| Fb. |  | 0.100 |  | 0.096 |  | 0.956 |  | 0.328 |  | 0.671 |  |
| Fb. + Age |  | 0.100 |  | 0.092 |  | 0.916 |  | 0.315 |  | 1.906 |  |
| Map. |  | 0.100 |  | 0.078 |  | 0.765 |  | 0.268 |  | 1.015 |  |
| Map. + Age |  | 0.100 |  | 0.071 |  | 0.688 |  | 0.243 |  | 3.093 |  |
| Map. + Fb.+ Map.x Fb. |  | 0.100 |  | 0.030 |  | 0.276 |  | 0.101 |  | 2.402 |  |
| Map. + Fb. + Age + Map. x Fb. |  | 0.100 |  | 0.026 |  | 0.240 |  | 0.089 |  | 2.284 |  |
| Map. + Fb. |  | 0.100 |  | 0.026 |  | 0.237 |  | 0.088 |  | 1.483 |  |
| Map. + Fb. + Age |  | 0.100 |  | 0.025 |  | 0.235 |  | 0.087 |  | 3.578 |  |
|  | | | | | | | | | | | |
| *Note.*  *All models include subject. Non-null models listed in order of BF_10_ . Map.: Color–Response_Mapping, Fb.: Feedback. BF_M_: Bayes factor of the change from prior to posterior model odds.* | | | | | | | | | | | |

**Omnibus regression to illustrate mapping-related change while testing the effect of cumulative performance feedback – Primary measure of outcome-sensitivity: NoGo accuracy**

Similar to Experiment 1, we derived a ΔNoGo_Accuracy DV to quantify the mapping-related change for each participant as the primary assay of outcome-sensitivity. We employed a hierarchical multiple regression model to extract the predictive strengths of the between-group regressors, Stim_Familiarity and Feedback, while controlling for Age, Gender, and Impulsivity. The resulting ΔR^2^ value for the contributions of Stim_Familiarity and Feedback determined whether mapping-related changes are predicted specifically by Stim_Familiarity (i.e., Familiar versus Novel stimuli), and whether the cumulative performance feedback manipulation plays a role in affecting motivational control. A corresponding *F*_change_ value was derived to confirm whether ΔR^2^ reflects a significant change in the model’s predictive strength. Participants with DV standardized residual values below -3.3 and above 3.3 were identified as outliers [4]. In such cases, we performed identical analyses without outlier participants to verify robustness of findings, but only report these excluded analyses if outliers produced substantial changes in statistical significance.

The regression model met the assumptions of normality and homoscedasticity. Multicollinearity tests produced negligible Variance Inflation Factors (VIF), confirming the assumption of linearity in the regression model (VIF for all variables < 1.04; see S9 Table).

Model 1, a linear combination of the controlled variables Age, Gender, and Impulsivity, did not significantly predict outcome-sensitivity: *F*(3,96) = 0.18, *p* = .91, and only explained 0.6% of the variance in ΔNoGo_Accuracy. Additionally, no controlled regressor independently predicted a change in ΔNoGo_Accuracy (all β coefficient *p’*s. > .05; see S9 Table). In the second step of the regression, the inclusion of the Stim_Familiarity and Feedback regressors explained an additional 14.5% of the variance in outcome-sensitivity: β_Stim_Familiarity_ = -.34, *p* = .001, β_Feedback_ = .18, *p* = .07, ΔR^2^ = .14, *F*_change_ (2,94) = 8.03, *p* = .001—rendering the entirety of Model 2 a significant predictor of outcome-sensitivity: *F*(5,94) = 3.34, *p* = .008 (see S9 Table).

We hypothesized that performance feedback may be a salient factor that can potentially restore goal-directed control when managing these well-established associations. However, cumulative performance feedback did not break the habits elicited by these familiar stimuli. As seen in the hierarchical multiple regression model, although Stim_Familiarity yielded differential mapping-related NoGo accuracy changes across these conditions, the Feedback regressor was not a significant predictor of outcome-sensitivity.

**S9 Table. Summary of the Hierarchical Multiple Regression Model for Outcome-Insensitivity as Assayed by ΔNoGo_Accuracy.**

| Variable | *Toler.* | *VIF* | *B* | *SE* | *β* | *t* | *sig.* |
| --- | --- | --- | --- | --- | --- | --- | --- |
| **Model 1** |  |  |  |  |  |  |  |
| Age | .99 | 1.01 | -0.31 | 0.58 | -.05 | -0.54 | .590 |
| Gender | .98 | 1.02 | -0.31 | 3.75 | -.01 | -0.08 | .935 |
| Impulsivity | .98 | 1.02 | 0.13 | 0.27 | .05 | 0.48 | .633 |
| **Model 2** |  |  |  |  |  |  |  |
| Age | .98 | 1.02 | -0.54 | 0.54 | -.09 | -1.00 | .319 |
| Gender | .96 | 1.04 | 1.10 | 3.53 | .03 | 0.31 | .756 |
| Impulsivity | .98 | 1.02 | 0.17 | 0.25 | .06 | 0.68 | .497 |
| Stim_Familiarity | .98 | 1.02 | -11.74 | 3.29 | -.34^**^ | -3.57 | **.001** |
| Feedback | .99 | 1.01 | 6.06 | 3.28 | .18 | 1.85 | .067 |

| Model Summary Statistics | | | | | | |
| --- | --- | --- | --- | --- | --- | --- |
| Model | *R^2^* | *F* | *F sig.* | *ΔR^2^* | *F_change_* | *F_change_ sig.* |
| **Model 1** | .01 | 0.18 | .910 |  |  |  |
| **Model 2** | .15 | 3.34 | **.008** | .14 | 8.03 | **.001** |

*Note: Top layer of table depicts all regressors included in the hierarchical model and their respective statistics. Bottom layer of table, Model Summary Statistics, depicts the predictive strength of each model. Delta R^2^ (ΔR^2^) and corresponding F_change_ values denote the specific improvement of Model 2 over Model 1 in predicting the dependent variable. Toler. = Tolerance; VIF = Variance Inflation Factor. Significant p-values (alpha = .05) depicted in bold typeface.*

**Bayesian analyses of the effects of performance feedback: Go accuracy**

The Bayes factor ANOVA of Familiar Stim_Familiarity condition Go accuracy illustrated that the data were 15.90 times more likely under the hypothesized Feedback x Color–Response_Mapping interaction model (BF_10_ = 15.90) over the null hypothesis. The best performing main effects model was Color–Response_Mapping, which was not substantially better fitting compared to the interaction model (BF_10_ = 16.63; 1.05 times more likely than the interaction model; see S10 Table).

**S10 Table. Bayesian analyses of the effects of performance feedback on Familiar Stim_Familiarity condition outcome-sensitivity: Go accuracy**

| **Model Comparison** | | | | | | | | | | | |
| --- | --- | --- | --- | --- | --- | --- | --- | --- | --- | --- | --- |
| **Models** | | **P(M)** | | **P(M\|data)** | | **BF _M_** | | **BF _10_** | | **error %** | |
| Null model (incl. subject) |  | 0.200 |  | 0.026 |  | 0.105 |  | 1.000 |  |  |  |
| Map. |  | 0.200 |  | 0.427 |  | 2.976 |  | 16.628 |  | 1.346 |  |
| Map. + Fb. + Map. x Fb. |  | 0.200 |  | 0.408 |  | 2.756 |  | 15.899 |  | 2.912 |  |
| Map. + Fb. |  | 0.200 |  | 0.132 |  | 0.610 |  | 5.158 |  | 2.161 |  |
| Fb/ |  | 0.200 |  | 0.008 |  | 0.030 |  | 0.293 |  | 0.948 |  |
|  | | | | | | | | | | | |
| *Note.*  *All models include subject. Non-null models listed in order of BF_10_ . Map.: Color–Response_Color–Response_Mapping, Fb.: Feedback.* *BF_M_: Bayes factor of the change from prior to posterior model odds.* | | | | | | | | | | | |

Our Bayesian analyses did not reveal substantial evidence for cumulative performance feedback affecting mapping-related change in Go accuracy when managing Novel associations. The associated Bayes factor ANOVA of Novel Stim_Familiarity Go accuracy did not identify the hypothesized Age-controlled Feedback x Color–Response_Mapping as a suitable fit for the data (BF_10_ = 0.20), as the data were 5 times more likely under the null hypothesis, and 5.81 times more likely under the best performing main effects model of Feedback (BF_10_ = 1.22) compared to the interaction model (see S11 Table).

**S11 Table. Bayesian analyses of the effects of performance feedback on Novel Stim_Familiarity condition outcome-sensitivity: Go accuracy**

| **Model Comparison** | | | | | | | | | | | |
| --- | --- | --- | --- | --- | --- | --- | --- | --- | --- | --- | --- |
| **Models** | | **P(M)** | | **P(M\|data)** | | **BF _M_** | | **BF _10_** | | **error %** | |
| Null model (incl. subject) |  | 0.100 |  | 0.181 |  | 1.990 |  | 1.000 |  |  |  |
| Fb. |  | 0.100 |  | 0.221 |  | 2.554 |  | 1.221 |  | 1.726 |  |
| Fb. + Age |  | 0.100 |  | 0.126 |  | 1.299 |  | 0.697 |  | 7.429 |  |
| Age |  | 0.100 |  | 0.120 |  | 1.225 |  | 0.662 |  | 1.475 |  |
| Map. + Fb. |  | 0.100 |  | 0.084 |  | 0.824 |  | 0.463 |  | 2.254 |  |
| Map. + Fb. + Map. x Fb. |  | 0.100 |  | 0.067 |  | 0.651 |  | 0.372 |  | 2.782 |  |
| Map. |  | 0.100 |  | 0.066 |  | 0.636 |  | 0.365 |  | 2.334 |  |
| Map. + Fb. + Age |  | 0.100 |  | 0.055 |  | 0.520 |  | 0.302 |  | 26.508 |  |
| Map. + Age |  | 0.100 |  | 0.041 |  | 0.389 |  | 0.229 |  | 1.150 |  |
| Map. + Fb. + Age + Map. x  Fb. |  | 0.100 |  | 0.039 |  | 0.361 |  | 0.213 |  | 6.610 |  |
|  | | | | | | | | | | | |
| *Note.*  *All models include subject. Non-null models listed in order of BF_10_ . Map.: Color–Response_Color–Response_Mapping, Fb.: Feedback.* *BF**_M_: Bayes factor of the change from prior to posterior model odds.* | | | | | | | | | | | |

**Omnibus regression to illustrate mapping-related change while testing the effect of cumulative performance feedback – Primary measure of outcome-sensitivity: Go accuracy**

We performed an identical omnibus hierarchical regression using ΔGo_Accuracy, which serves as our secondary measure of outcome-sensitivity. The regression model met the assumptions of normality and homoscedasticity. Multicollinearity tests produced negligible VIFs, confirming the assumption of linearity in the regression model (VIF for all variables < 1.04; see S12 Table). Two participants were identified as outliers due to a standardized residual values falling outside the predetermined range [4]. Identical analyses without outlier data produced no substantial changes in the statistical findings below.

Collectively, the linear combination of Age, Gender, and Impulsivity did not significantly predict mapping-related change in Go accuracy: *F*(3,96) = 0.31, *p* = .81. As depicted in S12 Table, closer examination of the individual regressors revealed no significant role played by any of the controlled variables in Model 1 (all β coefficient *p’*s. > .05; see S12 Table). In the second step of the regression, the inclusion of the Stim_Familiarity and Feedback regressors explained an additional 17.7% of the variance in Go accuracy change: β_Stim_Familiarity_ = -.32, *p* = .001, β_Feedback_ = .28, *p* = .003, ΔR^2^ = .18, *F*_change_(2,94) = 10.23, *p* < .001—rendering Model 2 a significant predictor of ΔGo_Accuracy: *F*(5,94) = 4.32, *p* = .001 (see S12 Table).

**S12 Table. Summary of the Hierarchical Multiple Regression Model for Outcome-Insensitivity as Assayed by ΔGo_Accuracy.**

| Variable | *Toler.* | *VIF* | *B* | *SE* | *β* | *t* | *sig.* |
| --- | --- | --- | --- | --- | --- | --- | --- |
| **Model 1** |  |  |  |  |  |  |  |
| Age | .99 | 1.01 | -0.11 | 0.21 | -.54 | -0.54 | .591 |
| Gender | .98 | 1.02 | -1.00 | 1.38 | -.07 | -0.73 | .469 |
| Impulsivity | .98 | 1.02 | -0.05 | 0.10 | -.05 | -0.53 | .600 |
| **Model 2** |  |  |  |  |  |  |  |
| Age | .98 | 1.02 | -0.22 | 0.20 | -.11 | -1.13 | .261 |
| Gender | .96 | 1.04 | -0.51 | 1.27 | -.04 | -0.40 | .692 |
| Impulsivity | .98 | 1.02 | -0.04 | 0.09 | -.04 | -0.43 | .670 |
| Stim_Familiarity | .98 | 1.02 | -4.03 | 1.19 | -.32^**^ | -3.39 | **.001** |
| Feedback | .99 | 1.01 | 3.59 | 1.18 | .28^**^ | 3.03 | **.003** |

| Model Summary Statistics | | | | | | |
| --- | --- | --- | --- | --- | --- | --- |
| Model | *R^2^* | *F* | *F sig.* | *ΔR^2^* | *F*_change_ | *F*_change_ *sig.* |
| **Model 1** | .01 | 0.31 | .815 |  |  |  |
| **Model 2** | .18 | 4.32 | **.001** | .18 | 10.23 | **<.001** |

*Note: Top layer of table depicts all regressors included in the hierarchical model and their respective statistics. Bottom layer of table, Model Summary Statistics, depicts the predictive strength of each model. Delta R^2^ (ΔR^2^) and corresponding F_change_ values denote the specific improvement of Model 2 over Model 1 in predicting the dependent variable. Toler. = Tolerance; VIF = Variance Inflation Factor. Significant p-values (alpha = .05) depicted in bold typeface.*

Although these hierarchical regression results regarding ΔGo_Accuracy suggest that cumulative performance feedback has significant predictive strength, the mixed-design ANOVAs in the main text indicate that cumulative performance feedback has a significant effect on Go actions only in the Familiar Stim_Familiarity condition (Novel Stim_Familiarity analysis interaction *p* = .05).

**Experiment 3**

**Replication of Experiment 1 via Bayesian analysis of outcome-sensitivity**

We followed up on each mixed-design ANOVA with Bayesian statistics. We derived BF_10_ for Stim_Familiarity and Color–Response_Mapping main effects and their interaction for each DV to replicate our findings from Experiment 1. We also performed similar Bayes factor ANOVAs to capture the role of cumulative performance feedback paired with a monetary bonus on mapping-related accuracy change. Thus, we examined our data for the potential suitability of the hypothesized Feedback x Color–Response_Mapping interaction model (controlling for any covariate that differed across groups) over models that solely included the main effects. We then divided the interaction’s BF_10_ by the main effects’ BF_10_ to quantify the likelihood of the interaction model fitting the data compared to the main effects’ models.

The Bayes factor ANOVA of NoGo accuracy that closely follows Experiment 1’s primary mixed-design ANOVA provided very strong evidence to support our replication of the Stim_Familiarity x Color–Response_Mapping interaction from Experiment 1. Specifically, we found that the Gender-corrected Stim_Familiarity x Color–Response_Mapping interaction model was 1367.36 times more likely to fit the data than the null hypothesis (BF_10_ = 1314.78). The best performing model was the non-corrected interaction model (BF_10_ = 3074.08; 2.33 times more likely than the Gender-corrected model; see S13 Table).

**S13 Table. Replication of Experiment 1 effect via Bayesian analyses of outcome-sensitivity: NoGo accuracy (No-Feedback group only).**

| **Model Comparison** | | | | | | | | | | | |
| --- | --- | --- | --- | --- | --- | --- | --- | --- | --- | --- | --- |
| **Models** | | **P(M)** | | **P(M\|data)** | | **BF _M_** | | **BF _10_** | | **error %** | |
| Null model (incl. subject) |  | 0.100 |  | 2.230e -4 |  | 0.002 |  | 1.000 |  |  |  |
| Map. + Stim_Fam. + Map. x Stim_Fam. |  | 0.100 |  | 0.685 |  | 19.613 |  | 3074.084 |  | 2.832 |  |
| Map. + Stim_Fam. + Gender + Map. x Stim_Fam. |  | 0.100 |  | 0.293 |  | 3.733 |  | 1314.785 |  | 2.666 |  |
| Map. + Stim_Fam. |  | 0.100 |  | 0.008 |  | 0.070 |  | 34.496 |  | 2.686 |  |
| Map. |  | 0.100 |  | 0.007 |  | 0.066 |  | 32.413 |  | 0.891 |  |
| Map. + Stim_Fam. + Gender |  | 0.100 |  | 0.003 |  | 0.028 |  | 13.927 |  | 2.244 |  |
| Map. + Gender |  | 0.100 |  | 0.003 |  | 0.025 |  | 12.309 |  | 2.067 |  |
| Stim_Fam. |  | 0.100 |  | 2.226e -4 |  | 0.002 |  | 0.999 |  | 0.903 |  |
| Stim_Fam. + Gender |  | 0.100 |  | 8.422e -5 |  | 7.580e -4 |  | 0.378 |  | 1.956 |  |
| Gender |  | 0.100 |  | 7.684e -5 |  | 6.916e -4 |  | 0.345 |  | 1.796 |  |
|  | | | | | | | | | | | |
| *Note.*  *All models include subject. Non-null models listed in order of BF_10_ . Map.: Color–Response_Mapping, Stim_Fam.: Stim_Familiarity.* *BF_M_: Bayes factor of the change from prior to posterior model odds.* | | | | | | | | | | | |

The Bayes factor ANOVA of Go accuracy provided some support for our hypotheses. We found that the Gender-corrected Stim_Familiarity x Color–Response_Mapping interaction model was 1.91 times more likely to fit the data than the null hypothesis (BF_10_ = 1.91), which was 1.45 times more likely than the non-corrected interaction (BF_10_ = 1.31; see S14 Table). Overall, we see slight Bayesian evidence for the interaction of the Stim_Familiarity and Color–Response_Mapping variables with Go accuracy as DV.

**S14 Table. Replication of Experiment 1 effect via Bayesian analyses of outcome-sensitivity: Go accuracy (No-Feedback group only).**

| **Model Comparison** | | | | | | | | | | | |
| --- | --- | --- | --- | --- | --- | --- | --- | --- | --- | --- | --- |
| **Models** | | **P(M)** | | **P(M\|data)** | | **BF _M_** | | **BF _10_** | | **error %** | |
| Null model (incl. subject) |  | 0.100 |  | 0.098 |  | 0.983 |  | 1.000 |  |  |  |
| Map. + Stim_Fam. + Gender+ Map, x Stim_Fam. |  | 0.100 |  | 0.188 |  | 2.085 |  | 1.910 |  | 3.227 |  |
| Gender |  | 0.100 |  | 0.147 |  | 1.546 |  | 1.489 |  | 1.159 |  |
| Map. + Stim_Fam. + Map. x Stim_Fam. |  | 0.100 |  | 0.129 |  | 1.336 |  | 1.312 |  | 3.069 |  |
| Map. + Gender |  | 0.100 |  | 0.122 |  | 1.252 |  | 1.240 |  | 1.617 |  |
| Map. |  | 0.100 |  | 0.084 |  | 0.821 |  | 0.849 |  | 3.064 |  |
| Stim_Fam. + Gender |  | 0.100 |  | 0.071 |  | 0.690 |  | 0.723 |  | 2.443 |  |
| Map. + Stim_Fam. + Gender |  | 0.100 |  | 0.062 |  | 0.596 |  | 0.630 |  | 2.907 |  |
| Stim_Fam. |  | 0.100 |  | 0.053 |  | 0.505 |  | 0.539 |  | 2.266 |  |
| Map. + Stim_Fam. |  | 0.100 |  | 0.046 |  | 0.430 |  | 0.463 |  | 2.664 |  |
|  | | | | | | | | | | | |
| *Note.*  *All models include subject. Non-null models listed in order of BF_10_ . Map.: Color–Response_Mapping, Stim_Fam.: Stim_Familiarity. BF_M_: Bayes factor of the change from prior to posterior model odds.* | | | | | | | | | | | |

**Bayesian analyses of the effects of dual feedback: NoGo accuracy**

The Bayes factor ANOVA of NoGo accuracy provided very strong evidence for the hypothesized Feedback x Color–Response_Mapping interaction model (BF_10_ = 9056.11), meaning the data were 9056.11 times more likely to occur under this interaction model compared to the null hypothesis. The next best performing model was the Mapping main effects model (BF_10_ = 1537.29), and the data were 5.89 times less likely to fit this model compared to the hypothesized interaction (see S15 Table).

**S15 Table. Bayesian analyses of the effects of dual feedback on Familiar Stim_Familiarity condition outcome-sensitivity: NoGo accuracy.**

| **Model Comparison** | | | | | | | | | | | |
| --- | --- | --- | --- | --- | --- | --- | --- | --- | --- | --- | --- |
| **Models** | | **P(M)** | | **P(M\|data)** | | **BF _M_** | | **BF _10_** | | **error %** | |
| Null model (incl. subject) |  | 0.200 |  | 8.472e -5 |  | 3.389e -4 |  | 1.000 |  |  |  |
| Map. + Fb. + Map. x Fb. |  | 0.200 |  | 0.767 |  | 13.184 |  | 9056.109 |  | 6.402 |  |
| Map. |  | 0.200 |  | 0.130 |  | 0.599 |  | 1537.286 |  | 0.671 |  |
| Map. + Fb. |  | 0.200 |  | 0.102 |  | 0.456 |  | 1208.663 |  | 1.734 |  |
| Fb. |  | 0.200 |  | 6.090e -5 |  | 2.436e -4 |  | 0.719 |  | 1.085 |  |
|  | | | | | | | | | | | |
| *Note.*  *All models include subject. Non-null models listed in order of BF_10_ . Map.: Color–Response_Mapping, Fb.: Feedback. BF_M_: Bayes factor of the change from prior to posterior model odds.* | | | | | | | | | | | |

The Bayes factor ANOVA did not provide evidence for the hypothesized Feedback x Color–Response_Mapping interaction model (controlled for Gender) when managing Novel Stim_Familiarity NoGo responses (BF_10_ = 0.19; moderate evidence for the null), meaning the data were 5.26 times less likely to fit this Gender-controlled interaction compared to the null hypothesis. The next best performing main effects model (excluding the controlled Gender model) was the Mapping model (BF_10_ = 0.65), and the data were 3.42 times more likely to fit this model compared to the hypothesized interaction (see S16 Table).

**S16 Table. Bayesian analyses of the effects of dual Feedback on Novel Stim_Familiarity condition outcome-sensitivity: NoGo accuracy.**

| **Model Comparison** | | | | | | | | | | | |
| --- | --- | --- | --- | --- | --- | --- | --- | --- | --- | --- | --- |
| **Models** | | **P(M)** | | **P(M\|data)** | | **BF _M_** | | **BF _10_** | | **error %** | |
| Null model (incl. subject) |  | 0.100 |  | 0.223 |  | 2.589 |  | 1.000 |  |  |  |
| Gender |  | 0.100 |  | 0.166 |  | 1.790 |  | 0.742 |  | 1.700 |  |
| Map. |  | 0.100 |  | 0.145 |  | 1.524 |  | 0.648 |  | 1.432 |  |
| Map. + Gender |  | 0.100 |  | 0.108 |  | 1.088 |  | 0.483 |  | 2.119 |  |
| Fb. |  | 0.100 |  | 0.091 |  | 0.898 |  | 0.406 |  | 0.757 |  |
| Fb. + Gender |  | 0.100 |  | 0.066 |  | 0.632 |  | 0.294 |  | 2.058 |  |
| Map. + Fb. + Map. x Fb. |  | 0.100 |  | 0.059 |  | 0.562 |  | 0.263 |  | 2.590 |  |
| Map. + Fb. |  | 0.100 |  | 0.058 |  | 0.557 |  | 0.261 |  | 1.214 |  |
| Map. + Fb. + Gender + Map. x Fb. |  | 0.100 |  | 0.043 |  | 0.402 |  | 0.191 |  | 2.810 |  |
| Map. + Fb. + Gender |  | 0.100 |  | 0.042 |  | 0.394 |  | 0.188 |  | 2.505 |  |
|  | | | | | | | | | | | |
| *Note.*  *All models include subject. Non-null models listed in order of BF_10_ . Map.: Color–Response_Mapping, Fb.: Feedback.* *BF_M_: Bayes factor of the change from prior to posterior model odds.* | | | | | | | | | | | |

**Bayesian analyses of the effects of dual feedback: Go accuracy**

The Bayes factor ANOVA of Feedback and Color–Response-Mapping of Familiar Go responses provided moderate evidence for the hypothesized Feedback x Color–Response_Mapping interaction model (BF_10_ = 5.43), meaning the data were 5.43 times more likely to occur under this interaction model compared to the null hypothesis. The next best performing model was the Feedback main effects model (BF_10_ = 3.04), and the data were 1.79 times less likely to fit this model compared to the hypothesized interaction (see S17 Table).

**S17 Table. Bayesian analyses of the effects of dual feedback on Familiar Stim_Familiarity condition outcome-sensitivity: Go accuracy**

| **Model Comparison** | | | | | | | | | | | |
| --- | --- | --- | --- | --- | --- | --- | --- | --- | --- | --- | --- |
| **Models** | | **P(M)** | | **P(M\|data)** | | **BF _M_** | | **BF _10_** | | **error %** | |
| Null model (incl. subject) |  | 0.200 |  | 0.071 |  | 0.307 |  | 1.000 |  |  |  |
| Map. + Fb. + Map. x Fb. |  | 0.200 |  | 0.387 |  | 2.525 |  | 5.431 |  | 2.538 |  |
| Fb. |  | 0.200 |  | 0.216 |  | 1.104 |  | 3.036 |  | 38.443 |  |
| Map. + Fb. |  | 0.200 |  | 0.211 |  | 1.072 |  | 2.966 |  | 1.674 |  |
| Map. |  | 0.200 |  | 0.114 |  | 0.515 |  | 1.600 |  | 0.879 |  |
|  | | | | | | | | | | | |
| *Note.*  *All models include subject. Non-null models listed in order of BF_10_ . Map.: Color–Response_Mapping, Fb.: Feedback. BF_M_: Bayes factor of the change from prior to posterior model odds.* | | | | | | | | | | | |

The Bayes factor ANOVA of Feedback and Color–Response-Mapping of Novel Go responses provided very strong evidence for the hypothesized Feedback x Color–Response_Mapping interaction model (BF_10_ = 104.58), meaning the data were 104.58 times more likely to occur under this interaction model compared to the null hypothesis. The next best performing model (excluding the interaction model without the Gender variable) was the Mapping main effects model (BF_10_ = 18.71), and the data were 5.59 times less likely to fit this model compared to the hypothesized interaction (see S18 Table).

**S18 Table. Bayesian analyses of the effects of dual Feedback on Novel Stim_Familiarity condition outcome-sensitivity: Go accuracy**

| **Model Comparison** | | | | | | | | | | | |
| --- | --- | --- | --- | --- | --- | --- | --- | --- | --- | --- | --- |
| **Models** | | **P(M)** | | **P(M\|data)** | | **BF _M_** | | **BF _10_** | | **error %** | |
| Null model (incl. subject) |  | 0.100 |  | 0.004 |  | 0.034 |  | 1.000 |  |  |  |
| Map. + Fb. + Map. x Fb. |  | 0.100 |  | 0.391 |  | 5.785 |  | 104.583 |  | 2.905 |  |
| Map. + Fb. + Gender + Map. x Fb. |  | 0.100 |  | 0.359 |  | 5.045 |  | 96.001 |  | 3.022 |  |
| Map. |  | 0.100 |  | 0.070 |  | 0.678 |  | 18.715 |  | 0.986 |  |
| Map. + Fb. |  | 0.100 |  | 0.060 |  | 0.573 |  | 15.993 |  | 2.999 |  |
| Map. + Fb. + Gender |  | 0.100 |  | 0.060 |  | 0.572 |  | 15.970 |  | 2.707 |  |
| Map. + Gender |  | 0.100 |  | 0.048 |  | 0.450 |  | 12.714 |  | 2.710 |  |
| Fb. |  | 0.100 |  | 0.003 |  | 0.029 |  | 0.871 |  | 3.979 |  |
| Fb. + Gender |  | 0.100 |  | 0.003 |  | 0.026 |  | 0.768 |  | 2.351 |  |
| Gender |  | 0.100 |  | 0.002 |  | 0.022 |  | 0.661 |  | 2.289 |  |
|  | | | | | | | | | | | |
| *Note.*  *All models include subject. Non-null models listed in order of BF_10_ . Map.: Color–Response_Mapping, Fb.: Feedback. BF_M_: Bayes factor of the change from prior to posterior model odds.* | | | | | | | | | | | |

**Omnibus regression to illustrate mapping-related change while testing the effect of dual feedback—cumulative performance and monetary feedback**

To detect the potential habit-disrupting effects of dual feedback (i.e., paired monetary and cumulative performance feedback), we employed a hierarchical multiple regression model to extract the predictive strengths of the between-group regressors, Stim_Familiarity and Feedback, while controlling for Age, Gender, and Impulsivity. We entered the controlled Age, Gender, and Impulsivity regressors into the first, and Stim_Familiarity and Feedback regressors of interest into the second step of the model to yield an R^2^ change (ΔR^2^) value that quantifies the contributions of Stim_Familiarity and Feedback. This allowed us to confirm whether mapping-related changes are predicted specifically by Stim_Familiarity (i.e., Familiar versus Novel stimuli), and whether dual feedback affected motivational control. A corresponding *F*_change_ value was derived to confirm whether ΔR^2^ reflects a significant change in the model’s predictive strength. Participants with DV standardized residual values below -3.3 and above 3.3 were identified as outliers [4]. In such cases, we performed identical analyses without outlier participants to verify robustness of findings, but only report these excluded analyses if outliers produced substantial changes in statistical significance.

The regression model met the assumptions of normality and homoscedasticity. Multicollinearity tests produced negligible Variance Inflation Factors (VIF), confirming linearity assumptions in the regression (VIF for all variables < 1.15; see S19 Table).

As hypothesized, Model 1, a linear combination of the controlled variables Age, Gender, and Impulsivity, did not significantly predict outcome-sensitivity: *F*(3,96) = 0.12, *p* = .95, and only explained 0.4% of the variance in ΔNoGo_Accuracy (R^2^ = .004). Additionally, none of these regressors independently predicted ΔNoGo_Accuracy (all β coefficient *p’*s > .05; see S19 Table). In the second step of the regression, the inclusion of the Stim_Familiarity and Feedback regressors explained an additional 26.6% of the variance in outcome-sensitivity: β_Stim_Familiarity_ = -.43, *p* < .001, β_Feedback_ = .28, *p* = .003, ΔR^2^ = .27, *F*_change_(2,94) = 17.16, *p* < .001—rendering Model 2 a significant predictor of outcome-sensitivity: *F*(5,94) = 6.96, *p* < .001 (see S19 Table).

**S19 Table. Summary of the Hierarchical Multiple Regression Model for Outcome-Insensitivity as Assayed by ΔNoGo_Accuracy.**

| Variable | *Toler.* | *VIF* | *B* | *SE* | *β* | *t* | *sig.* |
| --- | --- | --- | --- | --- | --- | --- | --- |
| **Model 1** |  |  |  |  |  |  |  |
| Age | .97 | 1.03 | 0.16 | 0.57 | .03 | 0.29 | .775 |
| Gender | .99 | 1.00 | 1.31 | 3.69 | .04 | 0.35 | .724 |
| Impulsivity | .97 | 1.03 | 0.09 | 0.22 | .04 | 0.40 | .692 |
| **Model 2** |  |  |  |  |  |  |  |
| Age | .94 | 1.06 | 0.24 | 0.50 | .04 | 0.48 | .635 |
| Gender | .87 | 1.14 | 0.14 | 3.41 | .004 | 0.04 | .968 |
| Impulsivity | .91 | 1.09 | 0.01 | 0.20 | .01 | 0.08 | .939 |
| Stim_Familiarity | .92 | 1.08 | -13.42 | 2.83 | -.43^***^ | -4.74 | **<.001** |
| Feedback | .89 | 1.13 | 8.76 | 2.89 | .28^**^ | 3.03 | **.003** |

| Model Summary Statistics | | | | | | |
| --- | --- | --- | --- | --- | --- | --- |
| Model | *R^2^* | *F* | *F sig.* | *ΔR^2^* | *F*_change_ | *F*_change_ *sig.* |
| **Model 1** | .004 | 0.12 | .950 |  |  |  |
| **Model 2** | .52 | 6.96 | **<.001** | .27 | 17.16 | **<.001** |

*Note: Top layer of table depicts all regressors included in the hierarchical model and their respective statistics. Bottom layer of table, Model Summary Statistics, depicts the predictive strength of each model. Delta R^2^ (ΔR^2^) and corresponding F_change_ values denote the specific improvement of Model 2 over Model 1 in predicting the dependent variable. Toler. = Tolerance; VIF = Variance Inflation Factor. Significant p-values (alpha = .05) depicted in bold typeface.*

As hypothesized, the delivery of cumulative performance and monetary feedback disrupted habits (i.e., prevent a significant incongruency-related change in NoGo accuracy to familiar lights) and improved goal-directed control (i.e., significantly increase NoGo accuracy to novel stimuli). In other words, these regression data suggest that the differential mapping-related NoGo change is replicated in Experiment 3, in that the stimulus Stim_Familiarity condition predicts changes in accuracy, and importantly, dual feedback is able to significantly predict improvements in performance.

*Secondary index of outcome-sensitivity: Go Accuracy*

We performed identical regression analyses using ΔGo_Accuracy as DV. The regression model met the assumptions of normality and homoscedasticity. Multicollinearity tests produced negligible VIFs, confirming the assumption of linearity in the regression model (VIF for all variables < 1.15). One participant was identified as an outlier due to a standardized residual value less than -3.3 [4]. Identical analyses without the outlier data produced no substantial changes in the statistical findings reported below; distinctions are specified where relevant.

Collectively, the linear combination of Age, Gender, and Impulsivity did not significantly predict mapping-related changes in Go accuracy: *F*(3,96) = 1.67, *p* = .18. As depicted in S20 Table, Model 1 only explained 5% of the variance (R^2^ = .05), and of all the controlled variables in Model 1, only the Impulsivity variable significantly predicted ΔGo_Accuracy: β_Impulsivity_ = .21, *p* = .04 (all other β coefficient *p’*s. > .05). In the second step of our regression model, the inclusion of the Stim_Familiarity and Feedback regressors explained an additional 21.7% of the variance in Go accuracy change: β_Stim_Familiarity_ = -.36, *p* < .001, β_Feedback_ = .28, *p* = .004, ΔR^2^ = .21, *F*_change_(2,94) = 13.11, *p* < .001—rendering Model 2 a significant predictor of ΔGo_Accuracy: *F*(5,94) = 6.50, *p* < .001 (see S20 Table). The predictive strength of the Impulsivity variable diminished below significance in Model 2 (β_Impulsivity_ = .17, *p* = .07). When reanalyzed without outlier data, Impulsivity did not predict ΔGo_Accuracy in either model (both *p*’s > .05). Given its lack of significant contributions in Experiments 1 and 2, and sensitivity to outlier correction in Experiment 3, we refrain from speculating further regarding the robustness of Impulsivity as a predictor.

**S20 Table. Summary of the Hierarchical Multiple Regression Model for Outcome-Insensitivity as Assayed by ΔGo_Accuracy.**

| Variable | *Toler.* | *VIF* | *B* | *SE* | *β* | *t* | *sig.* |
| --- | --- | --- | --- | --- | --- | --- | --- |
| **Model 1** |  |  |  |  |  |  |  |
| Age | .97 | 1.03 | 0.18 | 0.21 | .09 | 0.87 | .384 |
| Gender | .99 | 1.00 | 0.78 | 1.36 | .06 | 0.57 | .569 |
| Impulsivity | .97 | 1.03 | 0.17 | 0.08 | .21^*^ | 2.06 | **.042** |
| **Model 2** |  |  |  |  |  |  |  |
| Age | .94 | 1.06 | 0.20 | 0.19 | .09 | 1.03 | .306 |
| Gender | .87 | 1.14 | 0.55 | 1.30 | .04 | 0.42 | .673 |
| Impulsivity | .91 | 1.09 | 0.14 | 0.08 | .17 | 1.84 | .069 |
| Stim_Familiarity | .92 | 1.08 | -4.22 | 1.08 | -.36^***^ | -3.92 | **<.001** |
| Feedback | .89 | 1.13 | 3.25 | 1.10 | .28^**^ | 2.95 | **.004** |

| Model Summary Statistics | | | | | | |
| --- | --- | --- | --- | --- | --- | --- |
| Model | *R^2^* | *F* | *F sig.* | *ΔR^2^* | *F*_change_ | *F*_change_ *sig.* |
| **Model 1** | .05 | 1.67 | .178 |  |  |  |
| **Model 2** | .26 | 6.50 | **<.001** | .21 | 13.11 | **<.001** |

*Note: Top layer of table depicts all regressors included in the hierarchical model and their respective statistics. Bottom layer of table, Model Summary Statistics, depicts the predictive strength of each model. Delta R^2^ (ΔR^2^) and corresponding F_change_ values denote the specific improvement of Model 2 over Model 1 in predicting the dependent variable. Toler. = Tolerance; VIF = Variance Inflation Factor. Significant p-values (alpha = .05) depicted in bold typeface.*

The significant Stim_Familiarity and Feedback regressors indicate that in Experiment 3, Stim_Familiarity (Familiar vs. Novel) differentially yields changes in Go accuracy, and Feedback has a significant improvement effect on Go accuracy.

**References**

1. Jeffreys H. Theory of Probability. 3rd ed. London: Oxford University Press; 1961.

2. Lee MD, Wagenmakers E-J. Bayesian cognitive modeling: A practical course. New York, NY, US: Cambridge University Press; 2013. doi:10.1017/CBO9781139087759

3. Jarosz A, Wiley J. What Are the Odds? A Practical Guide to Computing and Reporting Bayes Factors. The Journal of Problem Solving. 2014;7. doi:10.7771/1932-6246.1167

4. Tabachnick BG, Fidell LS. Using multivariate statistics, 5th ed. Boston, MA: Allyn & Bacon/Pearson Education; 2007.
