# Supplementary material for: Demonstrating and disrupting well-learned habits
Source: PLoS One. 2020 Jun 12;15(6):e0234424. doi: 10.1371/journal.pone.0234424 (PMC7292414; doi:10.1371/journal.pone.0234424)
Supplement: S1 Data — (ZIP) [file pone.0234424.s002.zip › Habit_Disruption_Publish_Data/Experiment 1/Experiment1_Python_html_Output.html]

Experiment1\_Final\_Script


Experiment 1 Data. Stack data to facilitate plotting. Report NoGo and Go graphs. Exp 2 and 3 data found in their respective folders.

In [1]:

```
# import packages
import pandas as pd
import matplotlib as mpl
import matplotlib.pyplot as plt
import seaborn as sns
import numpy as np
from scipy import stats
from matplotlib.patches import Patch
%matplotlib inline
```

In [2]:

```
xls=pd.ExcelFile("Experiment1_Data_Final.xlsx")
df=pd.read_excel(xls, "Data_Clean")
df.head()
```

Out[2]:

|  | Subj\_ID | Stim\_Cond | Order | Go\_RT | Go\_Rev\_RT | Go\_ACC | Go\_Rev\_ACC | NoGo\_ACC | NoGo\_Rev\_ACC | NoGo\_Diff | Go\_Diff | BIS |
| --- | --- | --- | --- | --- | --- | --- | --- | --- | --- | --- | --- | --- |
| 0 | 1 | Familiar | RegFirst | 335.397727 | 315.988889 | 88 | 90 | 95.0 | 75.0 | -20.0 | 2 | 63 |
| 1 | 5 | Familiar | RegFirst | 322.927083 | 318.043956 | 96 | 91 | 90.0 | 90.0 | 0.0 | -5 | 65 |
| 2 | 9 | Familiar | RegFirst | 319.389474 | 312.139785 | 95 | 93 | 90.0 | 95.0 | 5.0 | -2 | 68 |
| 3 | 13 | Familiar | RegFirst | 325.272727 | 310.247191 | 88 | 89 | 60.0 | 55.0 | -5.0 | 1 | 73 |
| 4 | 17 | Familiar | RegFirst | 304.448718 | 308.848837 | 78 | 86 | 55.0 | 35.0 | -20.0 | 8 | 77 |

Congruency refers to the within-subject Mapping factor. In the Familiar condition, Congruent means Red:NoGo and Green:Go--congruent with daily experiences, whereas in the Novel condition, these congruency mappings are arbitrary: Purple:Go, Blue:NoGo.

In [3]:

```
stacked_data=pd.melt(df, id_vars=["Subj_ID", "Stim_Cond"], value_vars=["NoGo_ACC", "NoGo_Rev_ACC"], 
        var_name="Signal", value_name="Accuracy")
def conditions_phase(x):
    if x == "NoGo_ACC":
        return "Congruent"
    elif x == "NoGo_Rev_ACC":
        return "Incongruent"
func = np.vectorize(conditions_phase)
stacked_data["Congruency"] = func(stacked_data["Signal"])
stacked_data.head()
```

Out[3]:

|  | Subj\_ID | Stim\_Cond | Signal | Accuracy | Congruency |
| --- | --- | --- | --- | --- | --- |
| 0 | 1 | Familiar | NoGo\_ACC | 95.0 | Congruent |
| 1 | 5 | Familiar | NoGo\_ACC | 90.0 | Congruent |
| 2 | 9 | Familiar | NoGo\_ACC | 90.0 | Congruent |
| 3 | 13 | Familiar | NoGo\_ACC | 60.0 | Congruent |
| 4 | 17 | Familiar | NoGo\_ACC | 55.0 | Congruent |

In [4]:

```
sns.set(style="white", font="Times New Roman", context="notebook", font_scale=1.3)
ax = sns.barplot(x="Stim_Cond", y="Accuracy", hue="Congruency", palette=["#ff0000", "#03d547"], ci=68, capsize=0.02, data=stacked_data)
ax.patches[1].set_facecolor("#1d47f5")
ax.patches[3].set_facecolor("#d12fdf")
plt.title("Familiar and Novel stimuli: NoGo accuracy", weight="bold", y=1.08, fontsize=20)
plt.ylabel("NoGo Accuracy (%)", weight="bold", labelpad=5, fontsize=20)
plt.xticks(weight="bold", fontsize=20)
sns.despine(bottom=False)
ax.set_xlabel("")
ax.set_ylim(50,100)
ax.legend_.remove()
#add significance asterisk and line
x1, x2 = -0.20, 0.18   # only two columns, so they would be 0, 1
y, h, col = stacked_data['Accuracy'].mean()+30, 0.5, "k" #y will be the height of the line and star(2 points above the mean), h will be the height of the two lines
#pointing down--0.2, and col is the color--black coded as k 
plt.plot([x1, x1, x2, x2], [y, y+h, y+h, y], lw=1, c=col) #here we plot this line and star on top of our barplot
plt.text((x1+x2)*0.5, y+h-0.1, "*", ha='center', va='bottom', color=col)
plt.text((x1), 51, "Red", ha='center', va='bottom', color="white", size=20)
plt.text((x2+0.02), 51, "Green", ha='center', va='bottom', color="white", size=20)
plt.text((x2+0.62), 51, "Blue", ha='center', va='bottom', color="white", size=20)
plt.text((x2+1.02), 51, "Purple", ha='center', va='bottom', color="white", size=20)
plt.text(-0.2, 1.1, "A", weight="bold", fontsize=25, ha="left", va="bottom", transform=ax.transAxes)
#plt.savefig("Exp1_NoGo_graph_rev.tiff", bbox_inches="tight", dpi=300)
plt.show()
```

```
C:\Users\ahmet\Anaconda3\lib\site-packages\scipy\stats\stats.py:1713: FutureWarning: Using a non-tuple sequence for multidimensional indexing is deprecated; use `arr[tuple(seq)]` instead of `arr[seq]`. In the future this will be interpreted as an array index, `arr[np.array(seq)]`, which will result either in an error or a different result.
  return np.add.reduce(sorted[indexer] * weights, axis=axis) / sumval
```

In [5]:

```
stacked_data_go=pd.melt(df, id_vars=["Subj_ID", "Stim_Cond"], value_vars=["Go_ACC", "Go_Rev_ACC"], 
        var_name="Signal", value_name="Accuracy")
def conditions_phase(x):
    if x == "Go_ACC":
        return "Congruent"
    elif x == "Go_Rev_ACC":
        return "Incongruent"
func = np.vectorize(conditions_phase)
stacked_data_go["Congruency"] = func(stacked_data_go["Signal"])
stacked_data_go.head()
```

Out[5]:

|  | Subj\_ID | Stim\_Cond | Signal | Accuracy | Congruency |
| --- | --- | --- | --- | --- | --- |
| 0 | 1 | Familiar | Go\_ACC | 88 | Congruent |
| 1 | 5 | Familiar | Go\_ACC | 96 | Congruent |
| 2 | 9 | Familiar | Go\_ACC | 95 | Congruent |
| 3 | 13 | Familiar | Go\_ACC | 88 | Congruent |
| 4 | 17 | Familiar | Go\_ACC | 78 | Congruent |

In [6]:

```
sns.set(style="white", font="Times New Roman", context="notebook", font_scale=1.3)
ax = sns.barplot(x="Stim_Cond", y="Accuracy", hue="Congruency", palette=["#03d547", "#ff0000"], ci=68, capsize=0.02, data=stacked_data_go)
ax.patches[1].set_facecolor("#d12fdf")
ax.patches[3].set_facecolor("#1d47f5")
plt.title("Familiar and Novel stimuli: Go accuracy", weight="bold", y=1.08, fontsize=20)
plt.ylabel("Go Accuracy (%)", weight="bold", labelpad=5, fontsize=20)
plt.xticks(weight="bold", fontsize=20)
sns.despine(bottom=False)
ax.set_xlabel("")
ax.set_ylim(50,100)
ax.legend_.remove()
#add significance asterisk and line
x1, x2 = -0.20, 0.18   # only two columns, so they would be 0, 1
plt.text((x1), 51, "Green", ha='center', va='bottom', color="white", size=20)
plt.text((x2+0.02), 51, "Red", ha='center', va='bottom', color="white", size=20)
plt.text((x2+0.62), 51, "Purple", ha='center', va='bottom', color="white", size=20)
plt.text((x2+1.02), 51, "Blue", ha='center', va='bottom', color="white", size=20)
plt.text(-0.2, 1.1, "B", weight="bold", fontsize=25, ha="left", va="bottom", transform=ax.transAxes)
#plt.savefig("Exp1_Go_graph_rev.tiff", bbox_inches="tight", dpi=300)
plt.show()
```

```
C:\Users\ahmet\Anaconda3\lib\site-packages\scipy\stats\stats.py:1713: FutureWarning: Using a non-tuple sequence for multidimensional indexing is deprecated; use `arr[tuple(seq)]` instead of `arr[seq]`. In the future this will be interpreted as an array index, `arr[np.array(seq)]`, which will result either in an error or a different result.
  return np.add.reduce(sorted[indexer] * weights, axis=axis) / sumval
```
