# Supplementary material for: Demonstrating and disrupting well-learned habits
Source: PLoS One. 2020 Jun 12;15(6):e0234424. doi: 10.1371/journal.pone.0234424 (PMC7292414; doi:10.1371/journal.pone.0234424)
Supplement: S1 Data — (ZIP) [file pone.0234424.s002.zip › Habit_Disruption_Publish_Data/Experiment 3/Experiment3_Python_html_Output.html]

Experiment3\_Final\_Script


Experiment 3 data script. Melt data for graph creation.

In [1]:

```
#import all packages
import pandas as pd
import matplotlib as mpl
import matplotlib.pyplot as plt
import seaborn as sns
import numpy as np
from scipy import stats
from matplotlib.patches import Patch
%matplotlib inline
```

In [3]:

```
xls=pd.ExcelFile("Experiment3_Data_Final.xlsx")
df=pd.read_excel(xls, "Data_Clean")
pd.set_option("display.max_rows", 500)
df.head()
```

Out[3]:

|  | Subj\_ID | Stim\_Cond | FB\_Cond | Go\_RT | Go\_Rev\_RT | Go\_ACC | Go\_Rev\_ACC | NoGo\_ACC | NoGo\_Rev\_ACC | NoGo\_Diff | Go\_Diff | BIS |
| --- | --- | --- | --- | --- | --- | --- | --- | --- | --- | --- | --- | --- |
| 0 | 1 | Familiar | MonPerfFB | 286.824742 | 284.242105 | 97 | 95 | 90 | 95 | 5 | -2 | 78.0 |
| 1 | 2 | Familiar | MonPerfFB | 295.541667 | 283.655914 | 96 | 93 | 100 | 75 | -25 | -3 | 67.0 |
| 2 | 3 | Familiar | MonPerfFB | 307.096774 | 310.365591 | 93 | 93 | 65 | 85 | 20 | 0 | 67.0 |
| 3 | 4 | Familiar | MonPerfFB | 277.136842 | 277.041667 | 95 | 96 | 70 | 60 | -10 | 1 | 73.0 |
| 4 | 5 | Familiar | MonPerfFB | 299.291667 | 295.156250 | 96 | 96 | 95 | 90 | -5 | 0 | 70.0 |

Congruency refers to the within-subject Mapping factor. In the Familiar condition, Congruent means Red:NoGo and Green:Go--congruent with daily experiences, whereas in the Novel condition, these congruency mappings are arbitrary: Purple:Go, Blue:NoGo.

In [8]:

```
stacked_data=pd.melt(df, id_vars=["Subj_ID", "Stim_Cond", "FB_Cond"], value_vars=["NoGo_ACC", "NoGo_Rev_ACC"], 
                     var_name="Signal", value_name="Accuracy")
def condition_phase(x):
    if x == "NoGo_ACC":
        return "Congruent"
    elif x == "NoGo_Rev_ACC":
        return "Incongruent"
func = np.vectorize(condition_phase)
stacked_data["Congruency"] = func(stacked_data["Signal"])

stacked_data_fam = stacked_data.iloc[np.r_[0:50, 100:150]]

stacked_data_fam.head()
```

Out[8]:

|  | Subj\_ID | Stim\_Cond | FB\_Cond | Signal | Accuracy | Congruency |
| --- | --- | --- | --- | --- | --- | --- |
| 0 | 1 | Familiar | MonPerfFB | NoGo\_ACC | 90 | Congruent |
| 1 | 2 | Familiar | MonPerfFB | NoGo\_ACC | 100 | Congruent |
| 2 | 3 | Familiar | MonPerfFB | NoGo\_ACC | 65 | Congruent |
| 3 | 4 | Familiar | MonPerfFB | NoGo\_ACC | 70 | Congruent |
| 4 | 5 | Familiar | MonPerfFB | NoGo\_ACC | 95 | Congruent |

In [11]:

```
sns.set(style="white", context="notebook", font="Times New Roman", font_scale=1.3)
ax = sns.barplot(x="FB_Cond", y="Accuracy", hue="Congruency", palette=["#ff0000", "#03d547"], ci=68, capsize=0.02, data=stacked_data_fam)
plt.title("Familiar stimuli: NoGo accuracy", weight="bold", y=1.08, fontsize=20)
ax.set_ylim(50,100)
#ymajor = np.arange(50, 86, 5)
#ax.set_yticks(ymajor)
ax.set_xlabel("")
ax.set_ylabel("NoGo Accuracy (%)", weight="bold", labelpad=5, fontsize=20)
ax.set_xticklabels(["Feedback", "No Feedback"], fontsize=20)
ax.legend_.remove()
sns.despine(bottom=False)
#add sig asterisks and line
x1, x2 = -0.20, 0.18
x3, x4 = 0.79, 1.17
y, h, col = 98, 0.5, "k"
plt.plot([x3, x3, x4, x4], [y, y+h, y+h, y], lw=1, c=col)
plt.text((x3+x4)*0.5, y+h-0.1, "*", ha="center", va="bottom", color=col)
plt.text((x1), 51, "Red", ha='center', va='bottom', color="white", size=20)
plt.text((x2+0.02), 51, "Green", ha='center', va='bottom', color="white", size=20)
plt.text((x2+0.62), 51, "Red", ha='center', va='bottom', color="white", size=20)
plt.text((x2+1.02), 51, "Green", ha='center', va='bottom', color="white", size=20)
plt.text(-0.2, 1.1, "A", weight="bold", fontsize=25, ha="left", va="bottom", transform=ax.transAxes)
#plt.savefig("Exp7_NoGo_graph.tiff", bbox_inches="tight", dpi=300)
plt.show()
```

```
C:\Users\ahmet\Anaconda3\lib\site-packages\scipy\stats\stats.py:1713: FutureWarning: Using a non-tuple sequence for multidimensional indexing is deprecated; use `arr[tuple(seq)]` instead of `arr[seq]`. In the future this will be interpreted as an array index, `arr[np.array(seq)]`, which will result either in an error or a different result.
  return np.add.reduce(sorted[indexer] * weights, axis=axis) / sumval
```

In [12]:

```
stacked_data_nov = stacked_data.iloc[np.r_[50:100, 150:200]]

stacked_data_nov.head()
```

Out[12]:

|  | Subj\_ID | Stim\_Cond | FB\_Cond | Signal | Accuracy | Congruency |
| --- | --- | --- | --- | --- | --- | --- |
| 50 | 22 | Novel | MonPerfFB | NoGo\_ACC | 60 | Congruent |
| 51 | 23 | Novel | MonPerfFB | NoGo\_ACC | 80 | Congruent |
| 52 | 24 | Novel | MonPerfFB | NoGo\_ACC | 80 | Congruent |
| 53 | 25 | Novel | MonPerfFB | NoGo\_ACC | 55 | Congruent |
| 54 | 26 | Novel | MonPerfFB | NoGo\_ACC | 50 | Congruent |

In [13]:

```
sns.set(style="white", context="notebook", font="Times New Roman", font_scale=1.3)
ax = sns.barplot(x="FB_Cond", y="Accuracy", hue="Congruency", palette=["#1d47f5", "#d12fdf"], ci=68, capsize=0.02, data=stacked_data_nov)
plt.title("Novel stimuli: NoGo accuracy", weight="bold", y=1.08, fontsize=20)
ax.set_ylim(50,100)
ax.set_xlabel("")
ax.set_ylabel("NoGo Accuracy (%)", weight="bold", labelpad=5, fontsize=20)
ax.set_xticklabels(["Feedback", "No Feedback"], fontsize=20)
ax.legend_.remove()
sns.despine(bottom=False)
#add sig asterisks and line
x1, x2 = -0.20, 0.18
y, h, col = 98, 0.5, "k"
plt.text((x1), 51, "Blue", ha='center', va='bottom', color="white", size=20)
plt.text((x2+0.02), 51, "Purple", ha='center', va='bottom', color="white", size=20)
plt.text((x2+0.62), 51, "Blue", ha='center', va='bottom', color="white", size=20)
plt.text((x2+1.02), 51, "Purple", ha='center', va='bottom', color="white", size=20)
plt.text(-0.2, 1.1, "B", weight="bold", fontsize=25, ha="left", va="bottom", transform=ax.transAxes)
#plt.savefig("Exp4_NoGo_graph_Nov.tiff", bbox_inches="tight", dpi=300)
plt.show()
```

```
C:\Users\ahmet\Anaconda3\lib\site-packages\scipy\stats\stats.py:1713: FutureWarning: Using a non-tuple sequence for multidimensional indexing is deprecated; use `arr[tuple(seq)]` instead of `arr[seq]`. In the future this will be interpreted as an array index, `arr[np.array(seq)]`, which will result either in an error or a different result.
  return np.add.reduce(sorted[indexer] * weights, axis=axis) / sumval
```

In [15]:

```
stacked_data_go=pd.melt(df, id_vars=["Subj_ID", "Stim_Cond", "FB_Cond"], value_vars=["Go_ACC", "Go_Rev_ACC"], 
                     var_name="Signal", value_name="Accuracy")
def condition_phase(x):
    if x == "Go_ACC":
        return "Congruent"
    elif x == "Go_Rev_ACC":
        return "Incongruent"
func = np.vectorize(condition_phase)
stacked_data_go["Congruency"] = func(stacked_data_go["Signal"])

stacked_data_fam_go = stacked_data_go.iloc[np.r_[0:50, 100:150]]

stacked_data_fam_go.head()
```

Out[15]:

|  | Subj\_ID | Stim\_Cond | FB\_Cond | Signal | Accuracy | Congruency |
| --- | --- | --- | --- | --- | --- | --- |
| 0 | 1 | Familiar | MonPerfFB | Go\_ACC | 97 | Congruent |
| 1 | 2 | Familiar | MonPerfFB | Go\_ACC | 96 | Congruent |
| 2 | 3 | Familiar | MonPerfFB | Go\_ACC | 93 | Congruent |
| 3 | 4 | Familiar | MonPerfFB | Go\_ACC | 95 | Congruent |
| 4 | 5 | Familiar | MonPerfFB | Go\_ACC | 96 | Congruent |

In [16]:

```
sns.set(style="white", context="notebook", font="Times New Roman", font_scale=1.3)
ax = sns.barplot(x="FB_Cond", y="Accuracy", hue="Congruency", palette=["#03d547", "#ff0000"], ci=68, capsize=0.02, data=stacked_data_fam_go)
plt.title("Familiar stimuli: Go accuracy", weight="bold", y=1.08, fontsize=20)
ax.set_ylim(50,100)
ax.set_xlabel("")
ax.set_ylabel("Go Accuracy (%)", weight="bold", labelpad=5, fontsize=20)
ax.set_xticklabels(["Feedback", "No Feedback"], fontsize=20)
ax.legend_.remove()
sns.despine(bottom=False)
#add sig asterisks and line
x1, x2 = -0.20, 0.18
x3, x4 = 0.79, 1.17
y, h, col = 98, 0.5, "k"
plt.plot([x3, x3, x4, x4], [y, y+h, y+h, y], lw=1, c=col)
plt.text((x3+x4)*0.5, y+h-0.1, "*", ha="center", va="bottom", color=col)
plt.text((x1), 51, "Green", ha='center', va='bottom', color="white", size=20)
plt.text((x2+0.02), 51, "Red", ha='center', va='bottom', color="white", size=20)
plt.text((x2+0.62), 51, "Green", ha='center', va='bottom', color="white", size=20)
plt.text((x2+1.02), 51, "Red", ha='center', va='bottom', color="white", size=20)
plt.text(-0.2, 1.1, "C", weight="bold", fontsize=25, ha="left", va="bottom", transform=ax.transAxes)
#plt.savefig("Exp7_Fam_Go_graph.tiff", bbox_inches="tight", dpi=300)
plt.show()
```

```
C:\Users\ahmet\Anaconda3\lib\site-packages\scipy\stats\stats.py:1713: FutureWarning: Using a non-tuple sequence for multidimensional indexing is deprecated; use `arr[tuple(seq)]` instead of `arr[seq]`. In the future this will be interpreted as an array index, `arr[np.array(seq)]`, which will result either in an error or a different result.
  return np.add.reduce(sorted[indexer] * weights, axis=axis) / sumval
```

In [18]:

```
stacked_data_go=pd.melt(df, id_vars=["Subj_ID", "Stim_Cond", "FB_Cond"], value_vars=["Go_ACC", "Go_Rev_ACC"], 
                     var_name="Signal", value_name="Accuracy")
def condition_phase(x):
    if x == "Go_ACC":
        return "Congruent"
    elif x == "Go_Rev_ACC":
        return "Incongruent"
func = np.vectorize(condition_phase)
stacked_data_go["Congruency"] = func(stacked_data_go["Signal"])

stacked_data_nov_go = stacked_data_go.iloc[np.r_[50:100, 150:200]]

stacked_data_nov_go.head()
```

Out[18]:

|  | Subj\_ID | Stim\_Cond | FB\_Cond | Signal | Accuracy | Congruency |
| --- | --- | --- | --- | --- | --- | --- |
| 50 | 22 | Novel | MonPerfFB | Go\_ACC | 78 | Congruent |
| 51 | 23 | Novel | MonPerfFB | Go\_ACC | 84 | Congruent |
| 52 | 24 | Novel | MonPerfFB | Go\_ACC | 94 | Congruent |
| 53 | 25 | Novel | MonPerfFB | Go\_ACC | 89 | Congruent |
| 54 | 26 | Novel | MonPerfFB | Go\_ACC | 88 | Congruent |

In [19]:

```
sns.set(style="white", context="notebook", font="Times New Roman", font_scale=1.3)
ax = sns.barplot(x="FB_Cond", y="Accuracy", hue="Congruency", palette=["#d12fdf", "#1d47f5"], ci=68, capsize=0.02, data=stacked_data_nov_go)
plt.title("Novel stimuli: Go accuracy", weight="bold", y=1.08, fontsize=20)
ax.set_ylim(50,100)
ax.set_xlabel("")
ax.set_ylabel("Go Accuracy (%)", weight="bold", labelpad=5, fontsize=20)
ax.set_xticklabels(["Feedback", "No Feedback"], fontsize=20)
ax.legend_.remove()
sns.despine(bottom=False)
#add sig asterisks and line
x1, x2 = -0.20, 0.18
y, h, col = 98, 0.5, "k"
plt.plot([x1, x1, x2, x2], [y, y+h, y+h, y], lw=1, c=col)
plt.text((x1+x2)*0.5, y+h-0.1, "*", ha="center", va="bottom", color=col)
plt.text((x1), 51, "Purple", ha='center', va='bottom', color="white", size=20)
plt.text((x2+0.02), 51, "Blue", ha='center', va='bottom', color="white", size=20)
plt.text((x2+0.62), 51, "Purple", ha='center', va='bottom', color="white", size=20)
plt.text((x2+1.02), 51, "Blue", ha='center', va='bottom', color="white", size=20)
plt.text(-0.2, 1.1, "D", weight="bold", fontsize=25, ha="left", va="bottom", transform=ax.transAxes)
#plt.savefig("Exp7_Nov_Go_graph.tiff", bbox_inches="tight", dpi=300)
plt.show()
```

```
C:\Users\ahmet\Anaconda3\lib\site-packages\scipy\stats\stats.py:1713: FutureWarning: Using a non-tuple sequence for multidimensional indexing is deprecated; use `arr[tuple(seq)]` instead of `arr[seq]`. In the future this will be interpreted as an array index, `arr[np.array(seq)]`, which will result either in an error or a different result.
  return np.add.reduce(sorted[indexer] * weights, axis=axis) / sumval
```
